# Distinct Metabolomic Signatures in Preclinical and Obstructive Hypertrophic Cardiomyopathy

**DOI:** 10.3390/cells10112950

**Published:** 2021-10-29

**Authors:** Maike Schuldt, Beau van Driel, Sila Algül, Rahana Y. Parbhudayal, Daniela Q. C. M. Barge-Schaapveld, Ahmet Güçlü, Mark Jansen, Michelle Michels, Annette F. Baas, Mark A. van de Wiel, Max Nieuwdorp, Evgeni Levin, Tjeerd Germans, Judith J. M. Jans, Jolanda van der Velden

**Affiliations:** 1 Department of Physiology, Amsterdam Cardiovascular Sciences, Amsterdam UMC, Vrije Universiteit Amsterdam, 1081 HZ Amsterdam, The Netherlands; b.vandriel@amsterdamumc.nl (B.v.D.); s.algul@amsterdamumc.nl (S.A.); r.parbhudayal@amsterdamumc.nl (R.Y.P.); j.vandervelden1@amsterdamumc.nl (J.v.d.V.); 2 Department of Cardiology, Amsterdam UMC, Vrije Universiteit Amsterdam, 1081 HV Amsterdam, The Netherlands; a.guclu@isala.nl (A.G.); t.germans@amsterdamumc.nl (T.G.); 3 Department of Clinical Genetics, Leiden University Medical Center, 2300 RC Leiden, The Netherlands; D.Q.C.M.Barge-Schaapveld@lumc.nl; 4 Department of Cardiology, Isala Zwolle, 8025 AB Zwolle, The Netherlands; 5 Department of Genetics, University Medical Center Utrecht, Utrecht University, 3584 CX Utrecht, The Netherlands; M.Jansen-48@umcutrecht.nl (M.J.); A.F.Baas@umcutrecht.nl (A.F.B.); J.J.M.Jans@umcutrecht.nl (J.J.M.J.); 6 Department of Cardiology, Thorax Center, Erasmus Medical Center Rotterdam, 3015 GD Rotterdam, The Netherlands; m.michels@erasmusmc.nl; 7 Department of Epidemiology and Data Science, Amsterdam UMC, 1081 HV Amsterdam, The Netherlands; mark.vdwiel@amsterdamumc.nl; 8 Department of Internal and Vascular Medicine, Amsterdam UMC, Universiteit van Amsterdam, 1105 AZ Amsterdam, The Netherlands; m.nieuwdorp@amsterdamumc.nl (M.N.); e.levin@amsterdamumc.nl (E.L.)

**Keywords:** metabolomics, hypertrophic cardiomyopathy, disease signature, disease stage, serum, biomarker

## Abstract

Hypertrophic Cardiomyopathy (HCM) is a common inherited heart disease with poor risk prediction due to incomplete penetrance and a lack of clear genotype–phenotype correlations. Advanced imaging techniques have shown altered myocardial energetics already in preclinical gene variant carriers. To determine whether disturbed myocardial energetics with the potential to serve as biomarkers are also reflected in the serum metabolome, we analyzed the serum metabolome of asymptomatic carriers in comparison to healthy controls and obstructive HCM patients (HOCM). We performed non-quantitative direct-infusion high-resolution mass spectrometry-based untargeted metabolomics on serum from fasted asymptomatic gene variant carriers, symptomatic HOCM patients and healthy controls (*n* = 31, 14 and 9, respectively). Biomarker panels that discriminated the groups were identified by performing multivariate modeling with gradient-boosting classifiers. For all three group-wise comparisons we identified a panel of 30 serum metabolites that best discriminated the groups. These metabolite panels performed equally well as advanced cardiac imaging modalities in distinguishing the groups. Seven metabolites were found to be predictive in two different comparisons and may play an important role in defining the disease stage. This study reveals unique metabolic signatures in serum of preclinical carriers and HOCM patients that may potentially be used for HCM risk stratification and precision therapeutics.

## 1. Introduction

Hypertrophic cardiomyopathy (HCM) is the most common inherited heart disease, with a prevalence ranging from 1:500 to 1:200 [1,2]. A pathogenic gene variant can be found in ~50–60% of all patients, which means that family screening can be used to identify gene variant carriers (Carriers), who are at risk of developing cardiac disease, early in life. However, risk prediction is still poor due to incomplete penetrance and a lack of clear genotype–phenotype correlations [3]. Based on the current guidelines of the European Society of Cardiology (ESC), HCM is defined by a wall thickness of ≥13 mm in one or more left ventricular (LV) myocardial segments in first-degree family members of HCM patients [2]. To diagnose cardiac hypertrophy and dysfunction, the American College of Cardiology Foundation/American Heart Association recommends long-term clinical evaluations (echocardiography and electrocardiography) every 12–18 months from the age of 12 to 18–21 years, and at least every 5 years at ages of >21 years, while the ESC recommends routine clinical follow-up from the age of 10 every 1–2 years and from the age of 20 every 2–5 years.

Efforts have been made to find prognostic markers that can identify asymptomatic preclinical Carriers who are at risk of developing overt HCM. Ho and colleagues provided evidence for a pro-fibrotic response in Carriers without hypertrophy, illustrated by significantly elevated levels of the C-terminal pro-peptide of type I procollagen [4]. Furthermore, five out of six proteotypic peptides that were elevated in clinical HCM compared to controls were also significantly elevated in subclinical HCM in a study by Captur and colleagues [5]. Identification of blood biomarkers that reflect cardiac disease mechanisms at the preclinical disease stage may allow early treatment to prevent and/or delay disease.

Phosphorus-31 magnetic resonance spectroscopy showed an abnormal cardiac energetic state, evident from a decrease in the phosphocreatine to adenosine triphosphate ratio (PCr/ATP), in Carriers independently of the presence of hypertrophy [6]. In accordance, analysis of in vivo myocardial external efficiency (MEE) by combined ^11^C-acetate positron emission tomography (PET) and cardiovascular magnetic resonance (CMR) imaging showed increased myocardial oxygen consumption (MVO_2_) and decreased MEE in preclinical Carriers compared to healthy controls (Ctrls) [7,8]. At a cellular level, studies in human cardiac HCM samples showed that pathogenic gene variants increase myofilament calcium sensitivity, kinetics and tension cost and alter myosin sequestration [9,10,11,12,13,14], which may alter cardiomyocyte energetics and underlie the increase in MVO_2_. In patients with obstructive HCM (HOCM), a decrease in MVO_2_ can be observed compared to Ctrls and Carriers [15], which may be explained by secondary disease-related changes in metabolism and mitochondrial function in the hypertrophied myocardium. Indeed, proteomic analyses recently revealed lower levels of metabolic pathway proteins in myectomy samples from HOCM patients [16,17]. Overall, these studies show that metabolic changes (i.e., the energetic state of the heart) are central in the early (preclinical) and advanced disease stages of HCM and may have potential as biomarkers for disease stage-dependent diagnosis and treatment.

While advanced PET and CMR imaging to monitor energy status and metabolism of the heart is available, such imaging modalities are expensive and limited to specialized medical centers. Thus, there is an urgent need to find easily accessible serum biomarkers for improved risk prediction that can be tested in standard diagnostic laboratories. To determine whether changes in the metabolic serum profile are already evident at the preclinical stage of HCM, we performed an untargeted metabolomics screen in serum of asymptomatic Carriers in comparison to healthy Ctrls. To establish whether the metabolic serum profile is altered at the advanced disease stage, a comparison was made between Carriers and individuals with HOCM.

## 2. Materials and Methods

### 2.1. Study Population

The study protocol was in agreement with the principles outlined in the Declaration of Helsinki. Inclusion of individuals was determined as part of the Engine study [18], and approval for this study was given by the local Medical Ethics Review Committees (2004/226). Written informed consent was obtained from each individual prior to inclusion in this study. The study population consisted of 31 asymptomatic HCM-causing gene variant carriers without hypertrophy (Carriers) and 14 patients with symptomatic HOCM, defined according to the guidelines [2]. Exclusion criteria were: aortic stenosis, presence of coronary artery disease (coronary artery stenosis >30%), previous septal reduction therapy, poor LV function (ejection fraction <50%), a history of diabetes mellitus or hypertension (defined as a systemic blood pressure ≥140/90 mm Hg) and significant renal dysfunction defined as an estimated glomerular filtration rate <30 mL/min per 1.73∙m^2^ [15]. Table S1 provides an overview of the pathogenic variants of the Carriers and HOCM patients. The variants were identified using next-generation sequencing covering 48 cardiomyopathy-associated genes. Nine healthy variant-negative first-degree family members (age- and sex-matched) served as a control group (Ctrl). PET-CMR data for these groups have been published previously [7,18]. Clinical parameters of the different groups are shown in Table 1.

### 2.2. Blood Serum Preparation

Patients fasted overnight prior to collection of blood samples. The samples were collected by venous puncture and allowed to clot for 1 h at 37 °C. Following this incubation period, clotted samples were left to contract for at least 30 min at 4 °C. The serum was centrifuged (4000 rpm, 20 min, 4 °C) and separated. Sodium azide (0.01%) was added to the supernatant. All samples were stored at −20 °C until use.

### 2.3. Metabolomics Profiling

Metabolites were analyzed as described previously [19]. Briefly, a non-quantitative direct-infusion high-resolution mass spectrometry-based metabolomics method was performed in combination with a nano-electrospray ionization source. The mean peak intensities of the technical triplicates were calculated. Annotation was performed by matching the *m*/*z* of these mass peaks with a range of two parts per million to metabolite masses from the Human Metabolome Database [20]. Thus, mass peaks were identified.

### 2.4. Data Analysis and Modeling

Within the metabolomics data, we identified panels of metabolic biomarkers in order to discriminate between the following groups of subjects: Carrier vs. Ctrl, Carrier vs. HOCM and HOCM vs. Ctrl. In brief, we used a gradient-boosting classifier [21,22] to improve prediction accuracy. To train the model we used a fivefold stratified cross-validation over the data (80%), while the remaining data (20%) was used as the test dataset. We also applied a stability selection procedure [22] to ensure the robustness of the biomarker signatures. Finally, the receiver-operating characteristics area under the curve (AUROC) scores were computed and averaged. A permutation (randomization test) [23] was used to evaluate the statistical validity of the results. We built statistical models in Python v. 3.8 (www.python.org, accessed on 1 April 2019) with the packages Numpy, Scipy and Scikits-learn and applied R version 3.5.3 for visualizations with the functions ggplot2 and plotly.

### 2.5. Correlation Analysis

Pearson correlation with Benjamini–Hochberg correction was performed to check whether the top 30 metabolites individually correlated with clinical parameters.

### 2.6. Prediction Model

We used an additional prediction model to compare the performance of the clinical parameters MEE and MVO2 with the metabolomics data. The predictive performance of the clinical parameters MEE and MVO2was assessed with a standard logistic regression model as fit with R’s glm() function. As this is a low-dimensional model, predictions were simply obtained from the model fit. These probabilistic predictions defined the possible cut-offs for a positive test. Sensitivity and specificity were determined for these cut-offs and then visualized by the receiver-operating characteristics (ROC) curve. ROC and area under the ROC curve (AUC) were computed using R’s pROC package.

### 2.7. Analysis of Metabolite–Protein Links

For identification of functional links between blood metabolites and differentially expressed proteins in cardiac tissue from HOCM patients, the directly interacting enzymes of the top 30 metabolites of each pairwise comparison were identified using MetaBridge v1.2 based on the KEGG database. Subsequently, these metabolite-related proteins were matched with significantly different proteins in HOCM compared to controls derived from our previous proteomics study [17]. Additionally, we identified metabolite–protein links by manual literature search.

### 2.8. Statistics

GraphPad Prism v8 software was used for statistical analysis of clinical data. Clinical data in Table 1 were statistically analyzed using one-way ANOVA with Tukey’s multiple comparisons post hoc test or Chi-square test if appropriate. All values are shown as means ± the standard error of the mean. A *p*-value ≤ 0.05 was considered significant.

## 3. Results

### 3.1. Characteristics of Study Population

**Figure 1:**
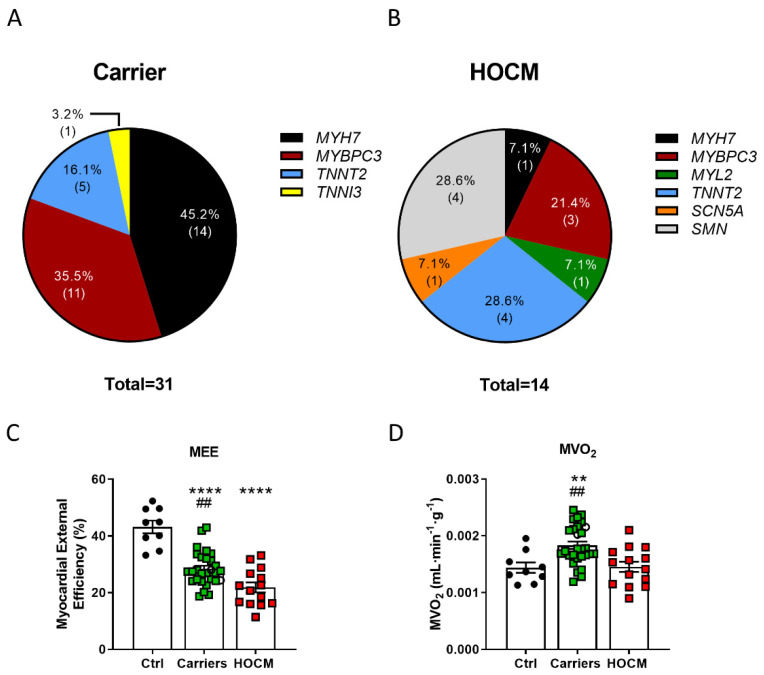
Proportions of affected genes and sarcomere mutation-negative (SMN) individuals in the (**A**) Carrier and (**B**) HOCM groups. (**C**) Myocardial external efficiency (MEE) and (**D**) myocardial oxygen consumption normalized to tissue weight (MVO_2_) in Ctrls, Carriers and HOCM patients (data have been published in Guclu et al. and Parbhudayal et al. [8,15]). **** *p* < 0.0001, ** *p* < 0.01 compared to Ctrls, ^##^ *p* < 0.01 compared to HOCM group, one-way ANOVA with Tukey’s multiple comparisons test.

Metabolomic serum profiling was performed in 31 Carriers, 14 HOCM patients and 9 Ctrls. Gene variants in the Carriers were present in *MYH7*, *MYBPC3* and genes encoding for troponins (Figure 1A; Table S1), whereas HOCM patients displayed genotypic variability, with 28.6% of them being sarcomere mutation-negative (Figure 1B). Figure 1A,B show an overview of the gene variants in our study groups. Figure S1 highlights the location of these variants. Patient characteristics are summarized in Table 1. The Carrier group was on average approximately 10 years younger than the HOCM and Ctrl groups and predominantly consisted of females, whereas HOCM patients and Ctrls included more males. The HOCM group showed significantly higher LV mass, a slightly increased LV ejection fraction and highly elevated NT-proBNP levels compared to Carriers and Ctrls, which is characteristic for the obstructive nature of the disease. Changes in the energetic status of the heart of included individuals, measured by PET-CMR imaging [8,15], are illustrated in Figure 1. Compared to Ctrls, Carriers showed increased MVO_2_, and both Carriers and HOCM patients showed reduced MEE (Figure 1C,D).

### 3.2. Multivariate Modeling Reveals Altered Metabolic Profile in Preclinical Stage of HCM

**Figure 2:**
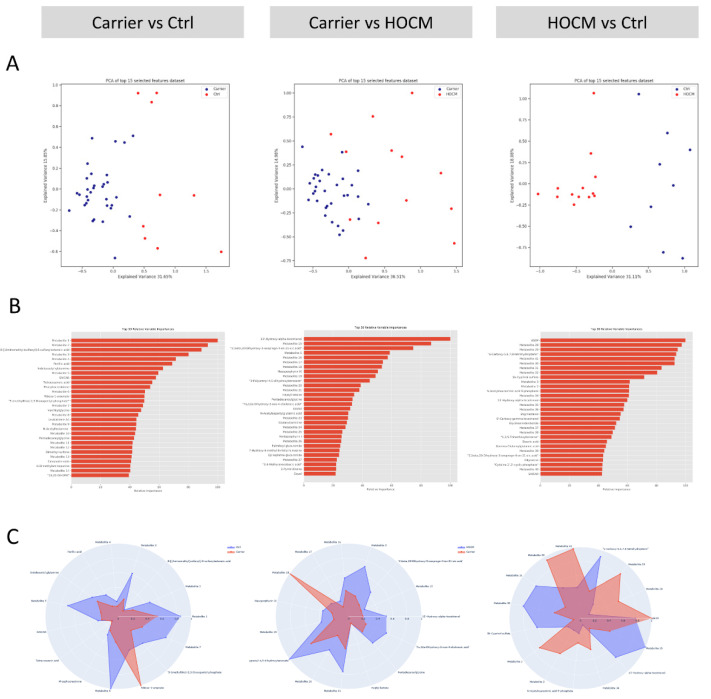
Multivariate modeling. (**A**) Principal component analysis (PCA) for the three group-wise comparisons: Carrier vs. Ctrls, Carrier vs. HOCM patients and HOCM patients vs. Ctrls. (**B**) Top 30 most predictive metabolites sorted based on their relative importance in distinguishing the two groups. (**C**) Radar plots of the top 15 most predictive metabolites, illustrating the differences in the serum metabolite profiles between the groups.

Multivariate modeling was performed for the Carrier vs. Ctrl groups, the Carrier vs. HOCM groups and the HOCM vs. Ctrl groups to determine whether the serum metabolome profiles could be used to distinguish the groups. The unsupervised principal component analysis (PCA) (Figure 2A), as well as the supervised partial least-squares discriminant analysis (PLS-DA, Figure S2A), which finds a linear regression model with categorical Y-variables, indeed showed distinct patterns in all group comparisons. Notably, the Carrier group clustered more tightly than the control and HOCM group, indicating less variability within the Carrier group compared to HOCM and Ctrl groups. The 30 most predictive metabolites for all three group comparisons are presented with their relative importance in Figure 2B, and their corresponding −log10(*p*) values are presented in Figure S2C. Some metabolites could not be uniquely identified because they shared the same mass with other metabolites. The possible identifications of these metabolites are listed in Table S2. The radar plots in Figure 2C show the 15 most important metabolites that differentiated the two respective groups and illustrate the differing serum metabolite profile in the different group comparisons. With this analysis we established metabolic signatures that define the three groups.

### 3.3. Distinct Metabolic Signatures at Preclinical and Symptomatic Stages of HCM

We listed and grouped the 30 most predictive metabolites of each comparison into different subgroups based on their chemical taxonomy super class (Table 2). The bar graphs with the individual data points are provided in Figure S3A–C for Carrier vs. Ctrl, Figure S4A–C for Carrier vs. HOCM and Figure S5A–C for HOCM vs. Ctrl (data from the third group are included in all comparisons). The numbers of metabolites per subgroup are shown in Table 2, illustrating that the majority of changes were in the “lipids and lipid-like molecules” and “organic acids and derivatives”.

Most metabolites that belonged to the category “organic acids and derivatives” in all three comparisons were dipeptides. The dipeptides differed between the different comparisons but represented a common finding. Lipids and lipid-like molecules were also among the top 30 metabolites in all three comparisons. Among these metabolites were fatty acids and conjugates, indicating changes in the fatty acid metabolism, but also eicosanoids, which are metabolites involved in the inflammatory response. The number of metabolites belonging to the category “lipids and lipid-like molecules” was largest in the comparisons with the HOCM group, suggesting that changes in lipid metabolism were predominant in the advanced disease stage.

**Figure 3:**
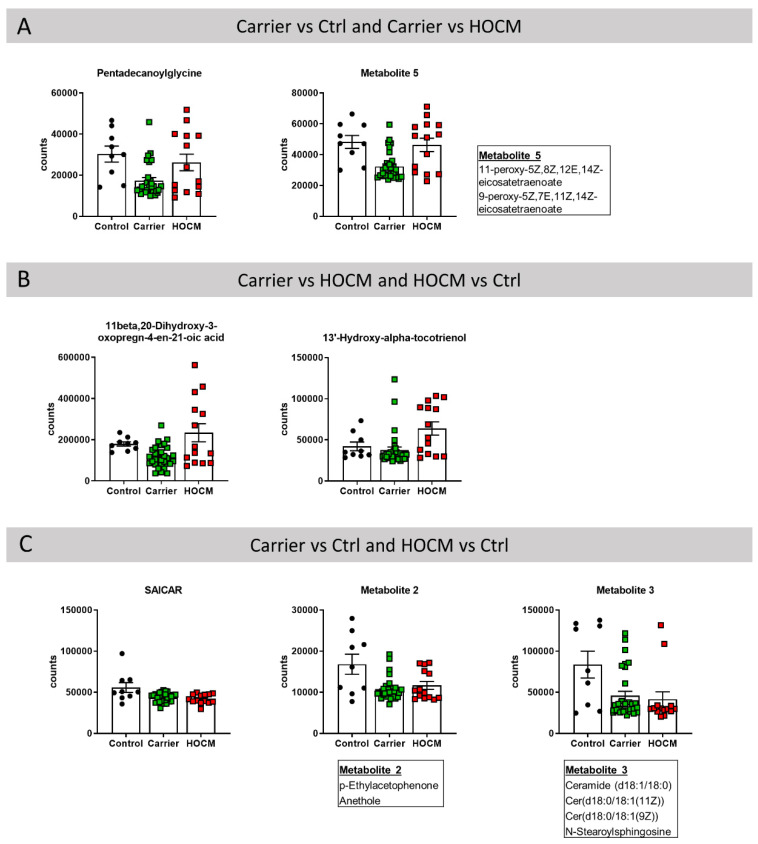
Metabolites that were among the 30 most important metabolites in distinguishing the groups in the (**A**) Carrier vs. Ctrl and Carrier vs. HOCM comparison, (**B**) Carrier vs. HOCM and HOCM vs. Ctrl comparison and (**C**) Carrier vs. Ctrl and HOCM vs. Ctrl comparison.

Notably, 7 of the 30 most important metabolites were predictive in two different comparisons (Figure 3). In this regard, pentadecanoylglycine and metabolite 5 were amongst the 30 most important metabolites in the Carrier vs. Ctrl comparison and the Carrier vs. HOCM comparison. This indicates that a decrease in these two metabolites may be an early change defining the Carrier group (Figure 3A). Levels of 11beta,20-dihydroxy-3-oxopregn-4-en-21-oic acid and 13′-hydroxy-alpha-tocotrienol were higher in the HOCM group and may therefore distinguish an advanced disease stage (Figure 3B). Among the 30 most important metabolites in the Carrier vs. Ctrl and the HOCM vs. Ctrl comparisons were SAICAR, metabolite 2 and metabolite 3. These metabolites showed lower levels in both the Carrier and the HOCM groups compared to Control and may characterize myocardial disease from early to late stages (Figure 3C).

Taken together, the seven metabolites that were predictive in two different comparisons may play an important role in defining disease stage.

To underline the biological relevance of the top 30 metabolites, we looked for direct links between these metabolites and significantly changed proteins in our recent proteomics study in myectomy tissue of HOCM patients [17]. We identified enzymes directly interacting with the metabolites using MetaBridge and overlapped these proteins with the significantly changed proteins from the HCM proteomics study. Overlapping proteins are reported in Table S3.

### 3.4. Metabolic Signature Versus In Vivo Energetic Status of the Heart

**Figure 4:**
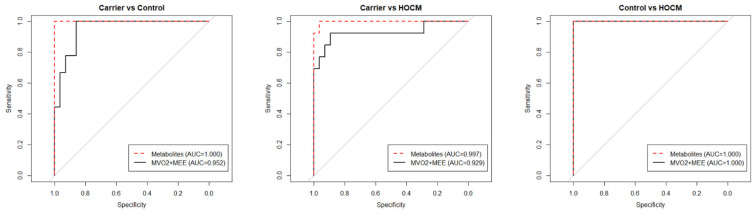
Sensitivity and specificity curves comparing the performances of the metabolomics data and the clinical imaging data in distinguishing the groups.

To determine whether the top metabolites reflected energetic changes in the heart, a correlation was made between metabolites and MEE and MVO_2_. Correlation analyses showed that 15 metabolites from all three sets of the top 30 metabolites significantly correlated with MEE, and 20 metabolites correlated with MVO_2_, but these correlations were not significant after correcting for multiple testing (*p* values are included in Table S2). This indicates that one single metabolite alone is not powerful enough as a predictive biomarker. To evaluate whether the complete metabolic signature could be used to identify preclinical Carriers who already show an altered energetic status of the heart, we compared the performance of the imaging parameters MEE and MVO_2_ with the metabolite signature in sensitivity and specificity curves (Figure 4). In all three group-wise comparisons the metabolite profile as well as the imaging parameters performed equally well in distinguishing the groups.

## 4. Discussion

In this study we investigated the ability of serum metabolites to discriminate preclinical carriers from healthy controls and HOCM patients. For each comparison we obtained a set of 30 metabolites that together were the most predictive in distinguishing these groups. Based on the number of metabolites per category in each comparison (Table 2), we hypothesize that changes in organic compounds are an early event in HCM pathogenesis and already present in asymptomatic carriers, while changes in lipid metabolism are pronounced at the advanced disease stage. Our study revealed unique metabolic signatures in the serum of Carriers and HOCM patients, which may have potential for risk stratification and precision therapeutics.

### 4.1. Altered Inflammatory Signature at Preclinical Disease Stage

Several inflammatory metabolites showed different expression patterns in the serum of Carriers compared to Ctrls or HOCM. The recent study by Captur et al. already showed that the complement C3-peptide is significantly elevated in subclinical and clinical HCM compared to controls and correlates with LV maximal wall thickness [5]. In line with this, Larson et al. have shown that levels of C3-peptide as well as other pro-inflammatory peptides decrease after surgical myectomy [24]. In this study, metabolite 2 (p-ethylacetophenone; anethole), which had lower levels in Carriers and HOCM patients, had anti-inflammatory properties that were mediated by either inhibition of production and/or release of inflammatory mediators, like tumor necrosis factor (TNF), nitric oxide, prostaglandins, interleukin 1 and interleukin 17 [25,26]. The anti-inflammatory effects of mesoporphyrin, which was lower in Carriers compared to HOCM patients, have been suggested to be mediated by inhibition of cytokine production, such as interferon-gamma and interleukin 6 [27]. Leukotrienes and prostaglandins, which can be summarized as eicosanoids, are more difficult to interpret since they can have pro-inflammatory as well as anti-inflammatory functions, which are dependent on the particular immunological context [28,29]. Metabolite 6 had leukotriene A4 as a possible metabolite identification and showed reduced levels in Carriers vs. Ctrls and intermediate levels in HOCM patients. Overall, the preclinical Carriers showed an altered inflammatory blood signature.

### 4.2. Altered Metabolic Blood Profile Reflects Changes in the HOCM Heart

The metabolic signatures in serum from Carriers and HOCM patients may have reflected disease mechanisms in the heart; the signature in Carriers may especially have reflected early disease changes. To validate the biological relevance of the identified metabolites in the context of HCM, we looked for common pathways and direct links between the top 30 metabolites and significantly changed proteins in our recent proteomics study on myectomy tissue of HOCM patients [17]. Indeed, several top metabolites could be linked to altered changes in the hearts of HOCM patients. Our recent proteomics analysis of 50 HOCM samples showed significantly lower levels of leukotriene A4 hydrolase, the enzyme that converts leukotriene A4 to leukotriene B4 [17]. The catalytic reaction inactivates the enzyme, which is observable in a change in molecular weight. Therefore, reduced levels of leukotriene A4 and the leukotriene A4 hydrolase could indicate increased conversion to leukotriene B4, which is implicated in inflammatory conditions [30]. Asymmetric dimethylarginine is directly linked to the enzyme dimethylarginine dimethylaminohydrolase 1. This enzyme is increased in myocardial tissue of HOCM patients and plays a role in nitric oxide generation, which is a key molecule in the process of inflammation [31].

As well as the inflammatory response, we also identified links between the serum metabolites and tissue proteins in the lipid and fatty acid metabolism. Tetracosanoic acid, a very-long-chain fatty acid, had lower serum levels in Carriers and HOCM patients compared to Ctrls. In tissue of HOCM patients we found reduced expression of long-chain fatty acid CoA ligase 1 [17] which catalyzes a step in the fatty acid beta-oxidation. Increased tissue protein levels of the long-chain fatty acid transport protein 6 [17], which translocates long-chain fatty acids across the plasma membrane [32], were in line with the elevated serum levels of stearic acid in HOCM patients compared to Ctrls. Reduced monoacylglycerol (metabolite 18 and 34) serum levels in HOCM patients compared to Ctrls with intermediate levels in Carriers were in line with the lower protein levels of monoglyceride lipase in the heart tissue of HOCM patients [17], which is the enzyme that can hydrolyze the reaction to fatty acids and glycerol. The metabolite glycerylphosphorylethanolamine is directly linked to lysophospholipase 1, which hydrolyzes fatty acids, and is decreased in the tissue of HCM patients. The top-30 metabolites indoleacetic acid and 5-hydroxyindoleacetaldehyde from the HOCM vs. Ctrl comparison had the aldehyde dehydrogenases ALDH9A1, ALDH3A2 and ALDH7A1 as directly interacting enzymes, of which ALDH3A2 was increased in HOCM tissue, whereas ALDH9A1 and ALDH7A1 had lower levels in HOCM patients. Since ALDH3A2 catalyzes the oxidation of medium- and long-chain aliphatic aldehydes to fatty acids, this finding also points towards an altered fatty acid metabolism.

As we can identify direct links between serum metabolites and protein changes in the heart, mainly regarding fatty acid metabolism, the top serum metabolites may reflect pathologic changes in the heart, underlining their biological relevance.

### 4.3. Disease Stage-Dependent Changes in Metabolic Signatures

A shift from fatty acids to glucose as the substrate for mitochondrial oxidation has been reported in acquired forms of cardiac hypertrophy and disease [33,34]. Acylcarnitines play a key role in transporting fatty acids across the mitochondrial membrane for subsequent fatty acid beta-oxidation [35] and lower acylcarnitine levels have been observed in the serum of asymptomatic or mildly symptomatic idiopathic HCM patients compared to controls in a study by Nakamura [36]. Changes in serum levels of fatty acids may therefore reflect substrate changes in the heart. One of the fatty acids elevated in HOCM patients compared to Carriers was glutarylcarnitine. This likely reflects levels of its precursor glutaryl-CoA, which is an intermediate of the lysine degradation pathway and feeds acetyl-CoA into the tricarboxylic acid (TCA) cycle. This potentially indicates an altered energetic demand.

**Figure 5:**
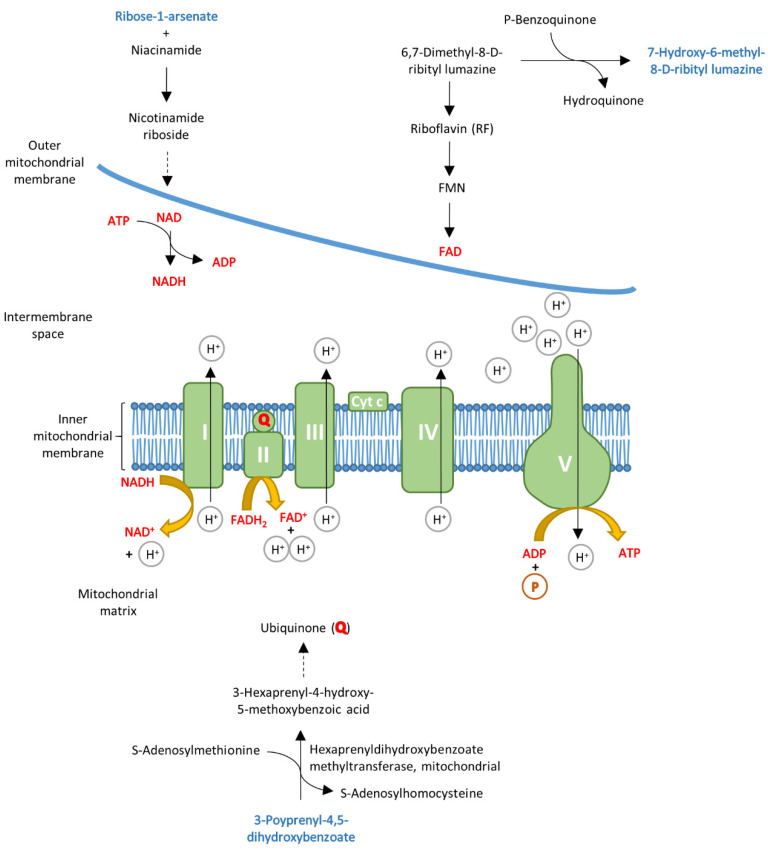
Schematic overview of the mitochondrial electron transport chain. Some of the top 30 metabolites (highlighted in blue) could be linked to energy metabolism, as they are involved in pathways that lead to essential molecules of the electron transport chain, which are highlighted in red.

Previous studies have shown different stages of energy deficiency in the myocardium of preclinical carriers and HOCM patients [15]. Interestingly, when comparing the serum from the Carrier and HOCM groups, we also found alterations of metabolites that could be linked to energy metabolism (highlighted in Figure 5). 7-Hydroxy-6-methyl-8-ribityl lumazine is an intermediate in the riboflavin metabolism that is important for building the enzyme cofactors flavin-adenine dinucleotide (FAD) and flavin mononucleotide (FMN) [37,38]. 3-Polyprenyl-4,5-dihydroxybenzoate, also elevated in the HOCM group, is a substrate for the mitochondrial enzyme hexaprenyldihydroxybenzoate methyltransferase, which is involved in the ubiquinone biosynthesis pathway and is important for the electron transport chain [39]. Carriers had elevated levels of the carbohydrate ribose-1-arsenate compared to controls. Ribose-1-arsenate is an intermediate in the arsenate detoxification pathway and is formed by the enzyme purine nucleoside phosphorylase [40]. We also found other components of the purine metabolism that were altered. Purine is not only a building block for the nucleotide bases adenosine and guanosine but also a component of the molecules ATP, GTP, cyclic AMP, NADH and coenzyme A, which are involved in cellular energy transmission. Although the metabolites are not directly involved in the electron transport chain, they are intermediates of pathways that lead to essential components of the electron transport chain (Figure 5).

### 4.4. Altered Protein Homeostasis in HCM

We found peptide alterations in both the carriers and the HOCM patients. Most of these peptides were dipeptides that were supposedly incomplete breakdown products of proteins. We can speculate that these changes were associated with the presence of mutant protein in carriers and HOCM patients causing increased protein degradation and synthesis to renew proteins. This would match findings of activated protein quality control in tissue of HOCM patients [41,42]. The dipeptide metabolite 11 (alanyl-leucine), which belongs to the branched-chain amino acids, was significantly elevated in carriers and HOCM patients compared to Ctrls. This was in line with previous findings of increased levels of branched-chain amino acids in a Finnish HCM cohort [43]. Interestingly, the first step in branched-chain amino acid catabolism occurs not in the liver but in the heart [44]. Hence, this may point to derailed branched-chain amino acid catabolism in the heart at the preclinical disease stage.

## 5. Conclusions

As shown in Figure 4, the serum metabolites performed as well as the advanced imaging modalities MEE and MVO_2_ in identifying preclinical Carriers and HOCM patients in our study cohort. Therefore, serum metabolites have the potential to serve as diagnostic biomarkers. As serum can be sampled repeatedly and with minimal invasiveness from individuals, a metabolite panel could be used to detect energetic changes in the heart to monitor carriers and determine the right time for therapeutic intervention. A recent study has shown that a plasma protein set can separate pre- and post-operative HCM patients undergoing septal myectomy [24]. The current untargeted metabolomics analysis shows that there are serum biomarker candidates that can, as a combined set, distinguish healthy controls, preclinical carriers with an altered energetic status of the heart and obstructive HCM patients. The findings in this study were derived from relatively small, heterogeneous groups with sex and age differences. Data about the dietary intake of the patients were not available; consequently, some of the metabolites may have been a result of differences in diet. While we here focused on blood biomarkers to detect early metabolic changes in the heart, follow-up metabolomics studies should also include ECG parameters of individuals, as previous studies have shown that electrophysiological abnormalities may appear before a clear echocardiographic pattern of hypertrophy [45,46].

The findings of this study provide a starting point for the ultimate goal of defining a set of blood biomarkers that is predictive for identifying asymptomatic gene variant carriers that will transition into a symptomatic state and are in need of preventive treatment. The top 30 metabolites that drove our multivariate model will need to be validated in follow-up studies with independent datasets, with the ultimate goal of defining a clinically feasible biomarker panel of four to six prognostic markers.

## Figures and Tables

**Table 1:** Demographic and clinical characteristics of the study population ^1^.

	Carrier (*n* = 31)	HOCM (*n* = 14)	Ctrl (*n* = 9)
**Age (years)**	38 ± 14 *	50 ± 13 ^#^	51 ± 8
**Male sex (no. (%)) ^$^**	7 (22.6)	10 (71.4)	6 (66.7)
**BMI (kg/m^2^)**	22.7 ± 2.9 *	26.9 ± 3.0 ^#^	26.4 ± 2.6
**LVM (g)**	72.4 ± 18.3	193.7 ± 68.7 *^,#^	102.5 ± 17.8
**LVM_i_**	39.6 ± 7.8	95.4 ± 33.0 *^,#^	49.5 ± 6.1
**LVEF (%)**	66 ± 6	71 ± 10 *	61 ± 6
**LVOTg (mmHg)**		34.7 ± 24.1	
**LVOTg (mmHg, post-myectomy)**		11 ± 8	
**Systolic BP (mmHg)**	113 ± 14	118 ± 17	124 ± 13
**Diastolic BP (mmHg)**	66 ± 9	69 ± 13	69 ± 4
**MAP (mmHg)**	81 ± 10	85 ± 14	88 ± 5
**HR (bpm)**	63 ± 11	60 ± 4	66 ± 9
**NT-proBNP (pg/l)**	77 ± 55 (*n* = 28)	1533 ± 2976 ^#^	54 ± 55
**FFA**	0.6 ± 0.2	0.3 ± 0.2^#^	0.5 ± 0.3

^1^ Values are given as means ± SD. Abbreviations: HOCM, hypertrophic obstructive cardiomyopathy; Ctrl, control; BMI, body mass index; LVM, left ventricular mass; LVMi, indexed LVM for body surface area; LVEF, left ventricular ejection fraction; LVOTg, left ventricular outflow tract gradient; BP; blood pressure; MAP, mean arterial pressure; HR, heart rate; FFA, free fatty acids. * *p* < 0.05 compared to Ctrl; ^#^ *p* < 0.05 compared to Carrier, one-way ANOVA with Tukey’s multiple comparisons test. ^$^ *p* < 0.05 Chi-square test. The Carrier group has three missing values for BMI and FFA, and the HOCM group has one missing value for FFA, five missing values for LVOTg and two missing values for LVOTg post-myectomy.

**Table 2:** Top 30 most important metabolites of the three pairwise comparisons categorized based on their chemical taxonomy super class ^2^.

Carrier vs. Ctrl	Carrier vs. HOCM	HOCM vs. Ctrl
Benzenoids		
2	1	2
**Metabolite 2**		**Metabolite 2**
Vanilloylglycine	3-Polyprenyl-4,5-dihydroxybenzoate	1,3,5-Trimethoxybenzene
Lipids and lipid-like molecules		
10	14	13
	**11beta,20-Dihydroxy-3-oxopregn-4-en-21-oic acid**	**11beta,20-Dihydroxy-3-oxopregn-4-en-21-oic acid**
	**13′-Hydroxy-alpha-tocotrienol**	**13′-Hydroxy-alpha-tocotrienol**
**Metabolite 3**		**Metabolite 3**
**Metabolite 5**	**Metabolite 5**	
8-[(Aminomethyl)sulfanyl]-6-sulfanyloctanoic acid	3,4-Methylenesebacic acid	5b-Cyprinol sulfate
19,20-DIHDPA	7a,12a-Dihydroxy-3-oxo-4-cholenic acid	9′-Carboxy-gamma-tocotrienol
Metabolite 1	Glutarylcarnitine	Metabolite 28
Metabolite 6	Metabolite 15	Metabolite 30
Metabolite 7	Metabolite 16	Metabolite 34
Metabolite 14	Metabolite 18	Metabolite 39
Perillic acid	Metabolite 22	Metabolite 40
Tetracosanoic acid	Metabolite 25	Metabolite 41
	Metabolite 26	Stearic acid
	Metabolite 27	Stigmastanol
	Palmitoyl glucuronide	
Nucleosides, nucleotides and analogues		
1	0	3
**SAICAR**		**SAICAR**
		dADP
		Glycineamideribotide
Organic acids and derivatives		
10	7	8
**Pentadecanoylglycine**	**Pentadecanoylglycine**	
5-(methylthio)-2,3-Dioxopentyl phosphate	Metabolite 17	Cytidine 2′,3′-cyclic phosphate
Indoleacetyl glutamine	Metabolite 19	Dityrosine
Metabolite 4	Metabolite 20	Gamma Glutamylglutamic acid
Metabolite 9	Metabolite 21	Metabolite 29
Metabolite 11	Metabolite 24	Metabolite 31
Metabolite 12	N-Acetylaspartylglutamic acid	Metabolite 32
Metabolite 13		Metabolite 36
N-Acetylhistamine		Metabolite 38
Phosphocreatinine		
Organic oxygen compounds		
3	2	2
Metabolite 8	Epinephrine glucoronide	Metabolite 33
Metabolite 10	Heptyl ketone	Metabolite 35
Ribose-1-arsenate		
Organoheterocyclic compounds		
2	5	2
6-Dimethylaminopurine	2-Pyrrolidinone	6-Carboxy-5,6,7,8-tetrahydropterin
Cinnavalininate	7-Hydroxy-6-methyl-8-ribityl lumazine	Metabolite 37
	Mesoporphyrin IX	
	Metabolite 23	
	Pentaporphyrin I	
Organosulfur compounds		
1	0	0
Dimethyl sulfone		
Phenylpropanoids and polyketides		
0	1	0
	Equol	

^2^ The numbers indicate the numbers of metabolites in the different metabolite categories. Metabolites that are significant in two pairwise comparisons are highlighted in bold.

## Data Availability

The data presented in this study are available on request from the corresponding author.

## Supplementary Materials

The following are available online at https://www.mdpi.com/article/10.3390/cells10112950/s1, Figure S1: Overview of gene variants in this study, Figure S2: Multivariate modeling, Figure S3: Bar graphs with individual data points for the top 30 most important metabolites in distinguishing the Carrier vs. Ctrl groups, Figure S4: Bar graphs with individual data points for the top 30 most important metabolites in distinguishing the Carrier vs. HOCM groups, Figure S5: Bar graphs with individual data points for the top 30 most important metabolites in distinguishing the HOCM vs. Ctrl groups, Table S1: Gene variants of preclinical carriers and HOCM patients, including the mutation type, sex and age of the individuals, Table S2: Metabolite identifications and functional classes of the top 30 metabolites from all three group-wise comparisons.
