# Supplementary material for: Distinct Metabolomic Signatures in Preclinical and Obstructive Hypertrophic Cardiomyopathy
Source: Cells. 2021 Oct 29;10(11):2950. doi: 10.3390/cells10112950 (PMC8616419; doi:10.3390/cells10112950)
Supplement: Supplementary file 1 [file cells-10-02950-s001.zip › cells-1315660-supplementary.pdf]

<u>Supplementary Material</u>

Table S1: Gene variants of preclinical carriers and HOCM patients including mutation type, sex and age of the individuals.

Gene	Mutation	Type, class	Age	Sex
**Preclinical Carriers**				
MYH7	c.1727A>G p.His576Arg	missense, class 4	34	F
MYH7	c.1207C>T p.Arg403Trp	missense, class 5	35	F
MYH7	c.1816G>A p.Val606Met	missense, class 4/5	30	M
MYH7	c.4130C>T p.Thr1377Met	missense, class 4	58	F
MYH7	c.4130C>T p.Thr1377Met	missense, class 4	36	F
MYH7	c.4130C>T p.Thr1377Met	missense, class 4	21	M
MYH7	c.1207C>T p.Arg403Trp	missense, class 5	21	F
MYH7	c.1207C>T p.Arg403Trp	missense, class 5	65	F
MYH7	c.4130C>T p.Thr1377Met	missense, class 4	44	F
MYH7	c.5135G>A p.Arg1712Gln	missense, class 4	45	F
MYH7	c.5135G>A p.Arg1712Gln	missense, class 4	33	M
MYH7	c.4130C>T p.Thr1377Met	missense, class 4	18	F
MYH7	c.4130C>T p.Thr1377Met	missense, class 4	31	F
MYBPC3	c.2373dup p.Trp792ValfsX41	frameshift, class 5	60	F
MYBPC3	c.2373dup p.Trp792ValfsX41	frameshift, class 5	28	F
MYBPC3	c.2373dup p.Trp792ValfsX41	frameshift, class 5	30	F

MYBPC3	c.2373dup p.Trp792ValfsX41	frameshift, class 5	22	M
MYBPC3	c.2373dup p.Trp792ValfsX41	frameshift, class 5	34	F
MYBPC3	c.2373dup p.Trp792ValfsX41	frameshift, class 5	60	F
MYBPC3	c.2373dup p.Trp792ValfsX41	frameshift, class 5	24	F
MYBPC3	c.2373dup p.Trp792ValfsX41	frameshift, class 5	56	F
MYBPC3	c.2373dup p.Trp792ValfsX41	frameshift, class 5	26	M
MYBPC3	c.2373dup p.Trp792ValfsX41	frameshift, class 5	43	F
MYBPC3	c.2373dup p.Trp792ValfsX41	frameshift, class 5	31	F
MYBPC3	c.897delG p.Lys301fs	frameshift, class 4/5	41	F
TNNT2	c.304C>T p.Arg102Trp	missense, class 5	59	F
TNNT2	c.304C>T p.Arg102Trp	missense, class 5	58	F
TNNT2	c.856C>T p.Arg286Cys	missense	48	F
TNNT2	c.430C>T p.Arg144Trp	missense, class 4/5	40	F
TNNT2	c.853C>T p.Arg285Cys	missense	32	M
TNNI3	c.497C>T p.Ser166Phe	missense, class 4/5	22	M
**HOCM patients**				
MYH7	p.Gln895His	missense	66	M
MYBPC3			54	F
MYBPC3	c.2373dup p.Trp792ValfsX41	frameshift, class 5	18	M
MYBPC3	c.2827C>T p.Arg943*	frameshift, class 5	54	F
TNNT2	c.853C>T	missense	43	M

	p.Arg285Cys			
TNNT2	c.304C>T p.Arg102Trp	missense, class 5	27	M
TNNT2	c.856C>T p.Arg286Cys	missense	53	M
TNNT2	c.835C>T p.Gln279*	truncation, class 4/5	54	M
MYL2	c.401A>C p.Glu134Ala	missense, class 3/4	57	F
Sarcomere mutation negative			64	M
Sarcomere mutation negative			52	M
Sarcomere mutation negative			68	F
Sarcomere mutation negative			46	M
Sarcomere mutation negative			47	M

Table S2: Metabolite identifications and functional classes of the top 30 metabolites of all three group-wise comparisons[1].

Metabolite	Possible identifications	Functional class	p value MEE	padj_BH MEE	p value MVO_2	padj_BH MVO_2
Metabolite 1	19-Hydroxyandrost-4-ene-3,17-dione; 19-Oxotestosterone; 7a-Hydroxyandrost-4-ene-3,17-dione; 11b-Hydroxyandrost-4-ene-3,17-dione; 16a-Hydroxyandrost-4-ene-3,17-dione; 4-Methoxy-17beta-estradiol; 2-Hydroxyestradiol-3-methyl ether	Lipids and lipid-like molecules				
**Metabolite 2**	p-Ethylacetophenone; Anethole	Benzenoids				
**Metabolite 3**	Ceramide (d18:1/18:0); Cer(d18:0/18:1(11Z)); Cer(d18:0/18:1(9Z)); N-Stearoylsphingosine	Lipids and lipid-like molecules	**0.0203**	0.9617		
Metabolite 4	Alanyl-Glutamine; Alanyl-Gamma-glutamate; Glutaminyl-Alanine; Gamma-glutamyl-Alanine; N-a-Acetylcitrulline	Organic acids and derivatives	**0.0226**	0.9617	**0.0039**	0.2924
**Metabolite 5**	11-peroxy-5Z,8Z,12E,14Z-eicosatetraenoate; 9-peroxy-5Z,7E,11Z,14Z-eicosatetraenoate	Lipids and lipid-like molecules			**0.0010**	0.2875
Metabolite 6	5-HEPE; Leukotriene A4; 12-HEPE; 14,15-EpETE; 15-<br>HEPE; 15-KETE; 17,18-EpETE; 5-KETE; 11R-HEPE; 18R-HEPE; 9-HEPE	Lipids and lipid-like molecules			**0.0010**	0.2875

Metabolite 7	Prostaglandin E3; Prostaglandin D3; 15-Keto-prostaglandin E2; Resolvin E1; 12-Oxo-20-hydroxy-leukotriene B4; 15-Epi-lipoxin B5; 15-Oxo-lipoxin A4; 20-oxo-leukotriene B4; PGH3; 5,12,18R-TriHEPE; 8-iso-15-keto-PGE2	Lipids and lipid-like molecules
Metabolite 8	4-Hydroxy-5-(3',5'-dihydroxyphenyl)-valeric acid-O-methyl-O-glucuronide; 4-Hydroxy-5-(3',4'-dihydroxyphenyl)-valeric acid-O-methyl-O-glucuronide	Organic oxygen compounds
Metabolite 9	Tryptophyl-Serine; Serinyl-Tryptophan	Organic acids and derivatives
Metabolite 10	O-6-deoxy-a-L-galactopyranosyl-(1->2)-O-b-D-galactopyranosyl-(1->4)-2-(acetylamino)-1,5-anhydro-2-deoxy-D-arabino-Hex-1-enitol; O-6-deoxy-a-L-galactopyranosyl-(1->2)-O-b-D-galactopyranosyl-(1->3)-2-(acetylamino)-1,5-anhydro-2-deoxy-D-arabino-Hex-1-enitol	Organic oxygen compounds
Metabolite 11	Alanyl-Leucine; Isoleucyl-Alanine; Leucyl-Alanine; Alanyl-Isoleucine	Organic acids and derivatives
Metabolite 12	Tyrosyl-Glutamate; Glutamyl-Tyrosine	Organic acids and derivatives
Metabolite 13	Glutamyl-Alanine; Alanyl-Glutamate	Organic acids and derivatives
Metabolite 14	sn-glycero-3-Phosphoethanolamine; Glycerylphosphorylethanolamine	Lipids and lipid-like molecules

Metabolite 15	S-Acetyldihydrolipoamide; S-Acetyldihydrolipoamide-E	Lipids and lipid-like molecules				
Metabolite 16	Medroxyprogesterone; 17-HDoHE; 19(20)-EpDPE; 16(17)-EpDPE; 10-HDoHE; 11-HDoHE; 16-HDoHE; 20-HDoHE; 4-HDoHE; 7-HDoHE; 8-HDoHE; 4-Hydroxy-all-trans-retinyl acetate	Lipids and lipid-like molecules			**0.0009**	0.2875
Metabolite 17	Aspartyl-Glutamate; Glutamyl-Aspartate	Organic acids and derivatives				
Metabolite 18	MG(0:0/20:3(5Z,8Z,11Z)/0:0); MG(0:0/20:3(8Z,11Z,14Z)/0:0); MG(20:3(11Z,14Z,17Z)/0:0/0:0); MG(20:3(5Z,8Z,11Z)/0:0/0:0); MG(20:3(8Z,11Z,14Z)/0:0/0:0); MG(0:0/20:3(11Z,14Z,17Z)/0:0)	Lipids and lipid-like molecules	**0.0012**	0.9617		
Metabolite 19	Hydroxyprolyl-Valine; Valyl-Hydroxyproline	Organic acids and derivatives				
Metabolite 20	Asparaginyl-Tryptophan; Histidinyl-Tyrosine; Tryptophyl-Asparagine; Tyrosyl-Histidine	Organic acids and derivatives				
Metabolite 21	Asymmetric dimethylarginine; Symmetric dimethylarginine	Organic acids and derivatives			**0.0045**	0.2924
Metabolite 22	Estriol; 2-Hydroxyestradiol; 16b-Hydroxyestradiol; 17-Epiestriol; 16,17-Epiestriol; 4-Hydroxyestradiol; 2-Polyprenyl-	Lipids and lipid-like molecules			**0.0014**	0.2875

	3-methyl-6-methoxy-1,4-benzoquinone; 4-hydroxystradiol					
Metabolite 23	Alpha-CEHC; Monoethylhexyl phthalic acid	Organoheterocyclic compounds			**<0.0001**	0.1720
Metabolite 24	Asparaginyl-Lysine; Lysyl-Asparagine	Organic acids and derivatives				
Metabolite 25	Tetracosahexaenoic acid; Tetracosahexaenoic acid, n-3	Lipids and lipid-like molecules			**0.0043**	0.2924
Metabolite 26	3'-Hydroxy-e,e-caroten-3-one; 3-Hydroxy-b,e-caroten-3'-one	Lipids and lipid-like molecules			**0.0076**	0.3950
Metabolite 27	15-Keto-13,14-dihydroprostaglandin A2; Prostaglandin J2; Prostaglandin A2; 12-Keto-leukotriene B4; Prostaglandin B2; Delta-12-Prostaglandin J2; Leukotriene B5; 5-Oxo-6-trans-leukotriene B4; 7'-Carboxy-gamma-chromanol; 15d PGD2; bicyclo-PGE2; Prostaglandin-c2	Lipids and lipid-like molecules			**0.0190**	0.5463
Metabolite 28	DG(16:0/15:0/0:0); DG(15:0/0:0/16:0); DG(15:0/16:0/0:0)	Lipids and lipid-like molecules				
Metabolite 29	Lysyl-Glutamine; Lysyl-Gamma-glutamate; Gamma-glutamyl-Lysine; Glutaminyl-Lysine	Organic acids and derivatives	**0.0107**	0.9617		
Metabolite 30	DG(15:0/14:0/0:0); DG(14:0/0:0/15:0); DG(14:0/15:0/0:0)	Lipids and lipid-like molecules			**0.0117**	0.4829

Metabolite 31	Cysteinyl-Leucine; Isoleucyl-Cysteine; Leucyl-Cysteine; Cysteinyl-Isoleucine	Organic acids and derivatives		
Metabolite 32	Tyrosyl-Glutamine; Tyrosyl-Gamma-glutamate; gln-tyr; Glutaminyl-Tyrosine	Organic acids and derivatives		
Metabolite 33	N-Acetyl-a-neuraminic acid; N-Acetylneuraminic acid	Organic oxygen compounds		
Metabolite 34	MG(20:5(5Z,8Z,11Z,14Z,17Z)/0:0/0:0); 9'-Carboxy-gamma-chromanol; MG(0:0/20:5(5Z,8Z,11Z,14Z,17Z)/0:0)	Lipids and lipid-like molecules	**0.0110**	0.9617
Metabolite 35	5-(3',4',5'-trihydroxyphenyl)-gamma-valerolactone-O-methyl-5'-O-glucuronide; 5-(3',4',5'-trihydroxyphenyl)-gamma-valerolactone-O-methyl-4'-O-glucuronide	Organic oxygen compounds	**0.0200**	0.9617
Metabolite 36	Tyrosyl-Cysteine; Cysteinyl-Tyrosine	Organic acids and derivatives		
Metabolite 37	5-Hydroxyindoleacetaldehyde; Indoleacetic acid			
Metabolite 38	Histidinyl-Threonine; Threoninyl-Histidine; 2-(3-Carboxy-3-aminopropyl)-L-histidine	Organic acids and derivatives		
Metabolite 39	DG(14:1(9Z)/20:0/0:0); DG(16:0/18:1(11Z)/0:0); DG(16:0/18:1(9Z)/0:0); DG(16:1(9Z)/18:0/0:0); DG(18:0/16:1(9Z)/0:0); DG(18:1(11Z)/16:0/0:0); DG(18:1(9Z)/16:0/0:0); DG(20:0/14:1(9Z)/0:0); DG(20:1(11Z)/14:0/0:0); DG(14:0/0:0/20:1n9);	Lipids and lipid-like molecules		

	DG(16:0/0:0/18:1n7); DG(16:0/0:0/18:1n9); DG(18:0/0:0/16:1n7); DG(20:0/0:0/14:1n5); DG(14:0/20:1(11Z)/0:0)					
Metabolite 40	Chenodeoxycholic acid 3-sulfate; Ursodeoxycholic acid 3-sulfate; Chenodeoxycholic acid sulfate	Lipids and lipid-like molecules				
Metabolite 41	MG(0:0/18:3(6Z,9Z,12Z)/0:0); MG(0:0/18:3(9Z,12Z,15Z)/0:0); MG(18:3(6Z,9Z,12Z)/0:0/0:0); MG(18:3(9Z,12Z,15Z)/0:0/0:0)	Lipids and lipid-like molecules	**0.0012**	0.9617		
1,3,5-Trimethoxybenzene		Benzenoids				
2-Pyrrolidinone		Organoheterocyclic compounds				
3-Polyprenyl-4,5-dihydroxybenzoate		Benzenoids			**0.0042**	0.2924
3,4-Methylenesebacic acid		Lipids and lipid-like molecules				
5-(methylthio)-2,3-Dioxopentyl phosphate		Organic acids and derivatives	**0.0013**	0.9617		
5b-Cyprinol sulfate		Lipids and lipid-like molecules				
6-Carboxy-5,6,7,8-tetrahydropterin		Organoheterocyclic compounds				

6-Dimethylaminopurine	Organoheterocyclic compounds		
7-Hydroxy-6-methyl-8-ribityl lumazine	Organoheterocyclic compounds		
7a,12a-Dihydroxy-3-oxo-4-cholenoic acid	Lipids and lipid-like molecules	**0.0003**	0.1957
8-[(Aminomethyl)sulfanyl]-6-sulfanyloctanoic acid	Lipids and lipid-like molecules		
9'-Carboxy-gamma-tocotrienol	Lipids and lipid-like molecules	**0.0486**	0.6320
**11beta,20-Dihydroxy-3-oxopregn-4-en-21-oic acid**	Lipids and lipid-like molecules	**0.0086**	0.4078
**13'-Hydroxy-alpha-tocotrienol**	Lipids and lipid-like molecules		
19,20-DiHDPA	Lipids and lipid-like molecules		
Cinnavalininate	Organoheterocyclic compounds		
Cytidine 2',3'-cyclic phosphate	Organic acids and derivatives		

dADP	Nucleosides, nucleotides and analogues	**0.0459**	0.9617		
Dimethyl sulfone	Organosulfur compounds				
Dityrosine	Organic acids and derivatives	**0.0208**	0.9617		
Epinephrine glucuronide	Organic oxygen compounds				
Equol	Phenylpropanoids and polyketides				
Gamma Glutamylglutamic acid	Organic acids and derivatives	**0.0447**	0.9617		
Glutarylcarnitine	Lipids and lipid-like molecules				
Glycineamideribotide	Nucleosides, nucleotides and analogues				
Heptyl ketone	Organic oxygen compounds				
Indoleacetyl glutamine	Organic acids and derivatives			**0.0469**	0.6225
Mesoporphyrin IX	Organoheterocyclic compounds				
N-Acetylaspartylglutamic acid	Organic acids and derivatives			**0.0251**	0.5533

N-Acetylhistamine	Organic acids and derivatives				
Palmitoyl glucuronide	Lipids and lipid-like molecules			**0.0239**	0.5533
**Pentadecanoylglycine**	Organic acids and derivatives			**0.0197**	0.5463
Pentaporphyrin I	Organoheterocyclic compounds	**0.0379**	0.9617		
Perillic acid	Lipids and lipid-like molecules				
Phosphocreatinine	Organic acids and derivatives				
Ribose-1-arsenate	Organic oxygen compounds				
**SAICAR**	Nucleosides, nucleotides and analogues	**0.0126**	0.9617		
Stearic acid	Lipids and lipid-like molecules				
Stigmastanol	Lipids and lipid-like molecules				
Tetracosanoic acid	Lipids and lipid-like molecules	**0.0119**	0.9617	**0.0318**	0.5549
Vanilloylglycine	Benzenoids	**0.0386**	0.9617		

[1]Metabolites in bold are significant in 2 comparisons. P value is given for metabolites that correlate significantly with myocardial external efficiency (MEE) or myocardial oxygen consumption (MVO_2), together with the adjusted p value for multiple testing.

Table S3: Metabolite-Protein links identified with MetaBridge.

Carrier vs Ctrl	Carrier vs HOCM	HOCM vs Ctrl
LTA4H	DDAH1	ALDH9A1
LYPLA1		ALDH3A2
		ALDH7A1
		GART

A

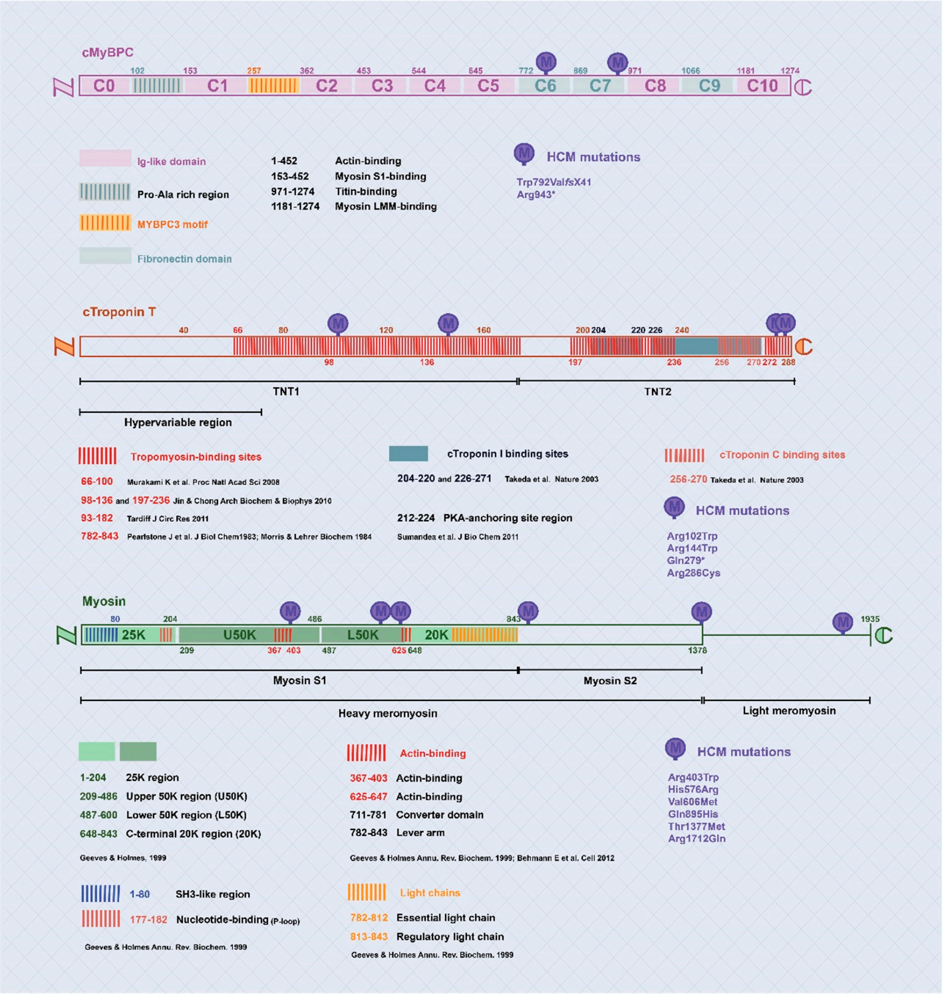

B

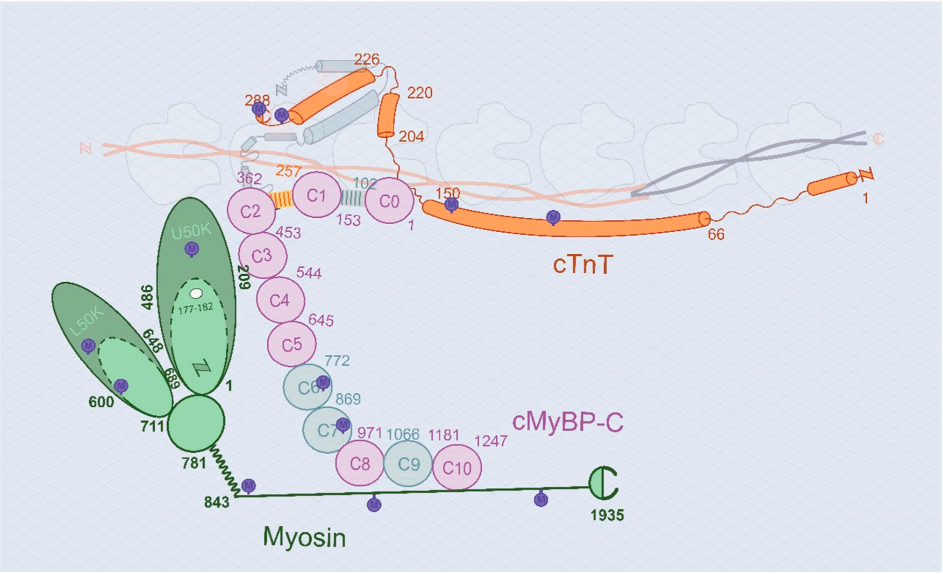

Figure S1: Overview of gene variants in this study. (A) shows the protein domains in which the gene variants are located. Image with permission granted by Vasco Sequeira. (B) indicates the location of the gene variants in the structural context. Image adapted from Sequeira et al. Circ Res 2013 with permission Circ Res.

Figure S2

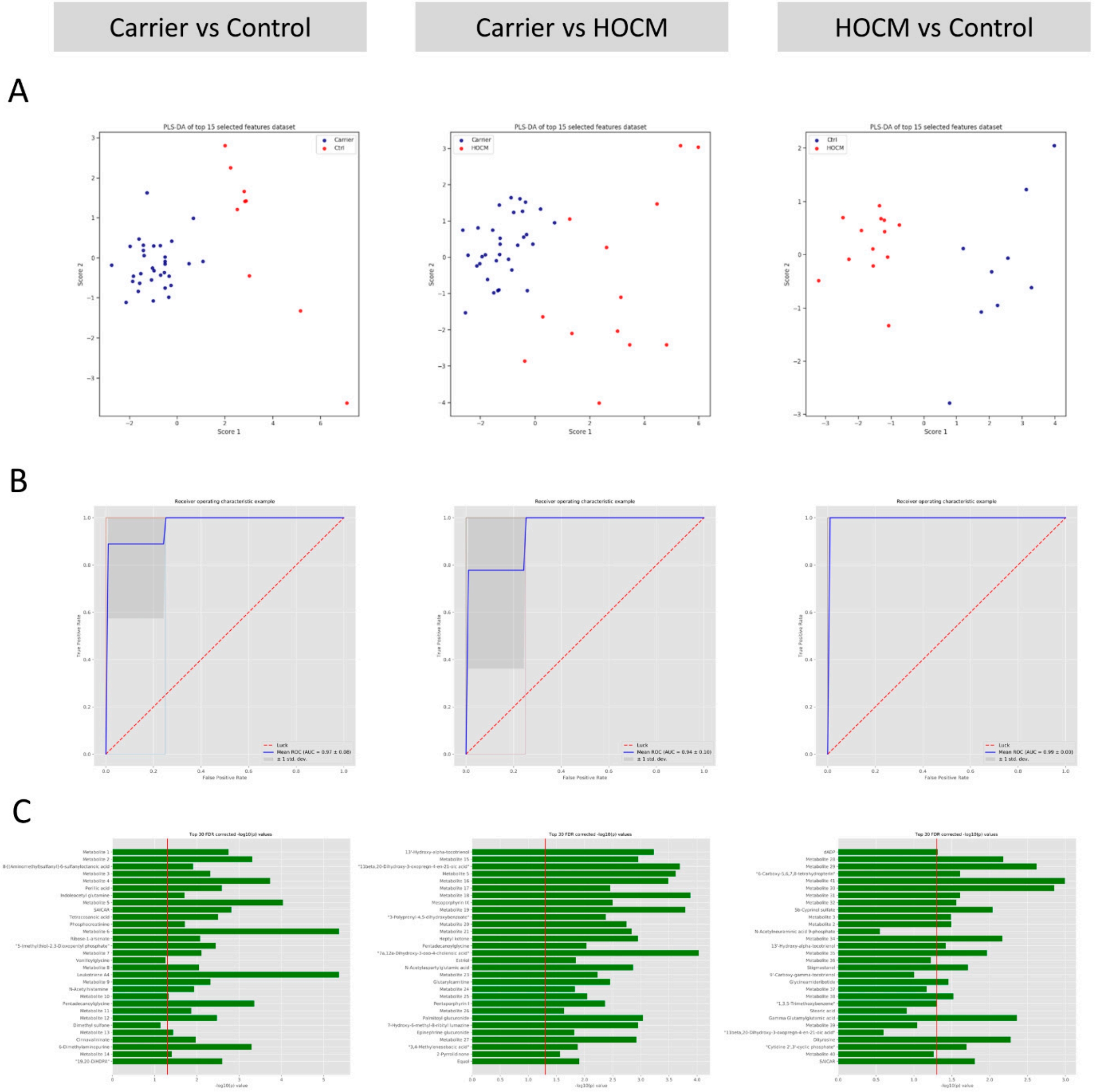

Figure S2: Multivariate modelling. (A) Partial least square-discriminant analysis plots (PLS-DA) for the three group-wise comparisons Carrier vs Ctrls, Carrier vs HOCM and HOCM vs Ctrls. (B) ROC curves of the three group-wise comparisons presenting the performance of the metabolomics data in distinguishing the groups. (C) Top 30 most predictive metabolites with corresponding –log10(p)-values.

Carrier vs Ctrl

Organic acids and derivatives

Amino acids, peptides and analogues

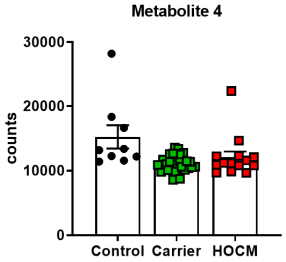

Metabolite 4
Alanyl-Glutamine
Alanyl-Gamma-glutamate
Glutaminyl-Alanine
Gamma-glutamyl-Alanine
N-a-Acetylcitrulline

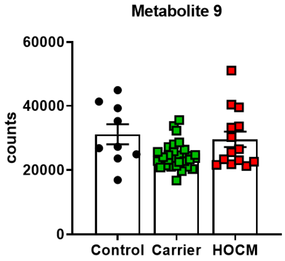

Metabolite 9
Tryptophyl-Serine
Serinyl-Tryptophan

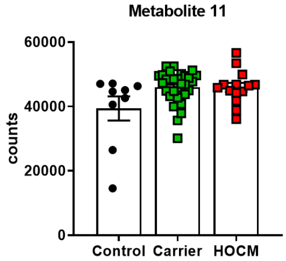

Metabolite 11
Alanyl-Leucine
Isoleucyl-Alanine
Leucyl-Alanine
Alanyl-Isoleucine

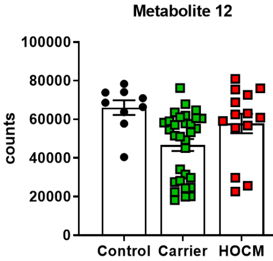

Metabolite 12
Tyrosyl-Glutamate
Glutamyl-Tyrosine

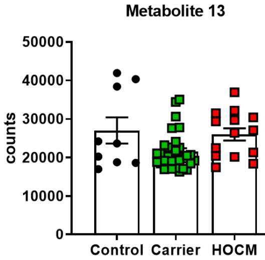

Metabolite 13
Glutamyl-Alanine
Alanyl-Glutamate

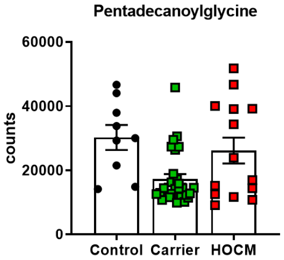

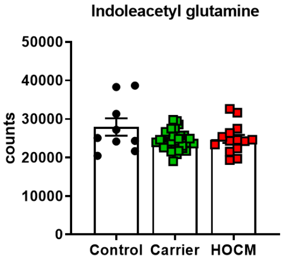

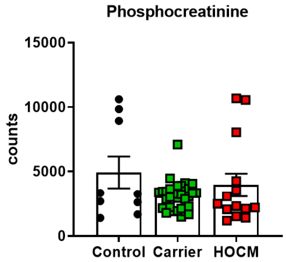

Phosphate esters

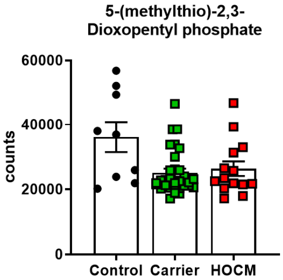

Carboxylic acid derivatives

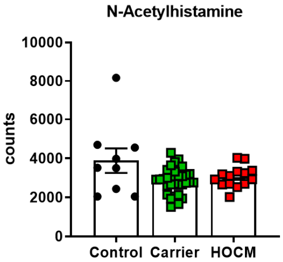

Carrier vs Ctrl

Lipids and lipid-like molecules

Fatty acids and conjugates

Metabolite 5

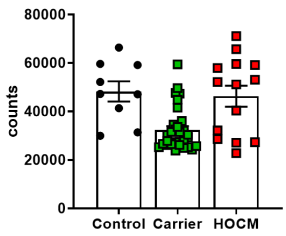

Metabolite 5
11-peroxy-5Z,8Z,12E,14Z-eicosatetraenoate
9-peroxy-5Z,7E,11Z,14Z-eicosatetraenoate

Metabolite 6

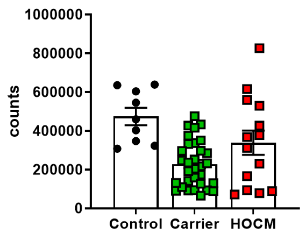

Metabolite 6
5-HEPE
Leukotriene A4
12-HEPE
14,15-EpETE
15-HEPE
15-KETE
17,18-EpETE
5-KETE
11R-HEPE
18R-HEPE
9-HEPE

8-[(Aminomethyl)sulfanyl]-6-sulfanyloctanoic acid

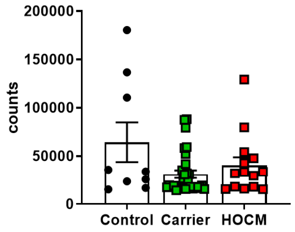

Tetracosanoic acid

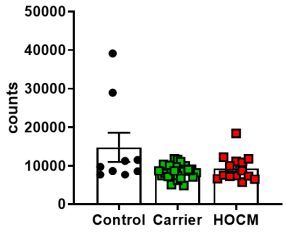

19,20-DiHDPA

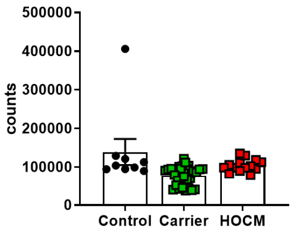

Ceramides

Metabolite 3

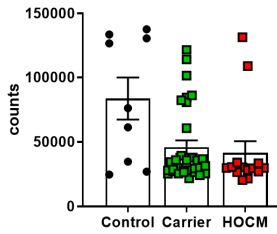

Metabolite 3
Ceramide (d18:1/18:0)
Cer(d18:0/18:1(11Z))
Cer(d18:0/18:1(9Z))
N-Stearoylsphingosine

Eicosanoids

Metabolite 7

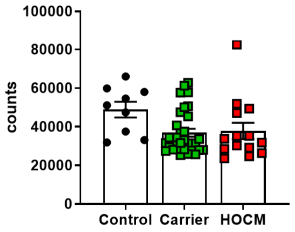

Metabolite 7
Prostaglandin E3
Prostaglandin D3
15-Keto-prostaglandin E2
Resolvin E1
12-Oxo-20-hydroxy-leukotriene B4
15-Epi-lipoxin B5
15-Oxo-lipoxin A4
20-oxo-leukotriene B4
PGH3
5,12,18R-TriHEPE
8-iso-15-keto-PGE2

Monoterpenoids

Perillic acid

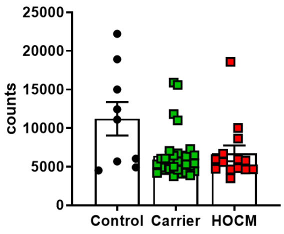

Glycerophosphoethanolamines

Metabolite 14

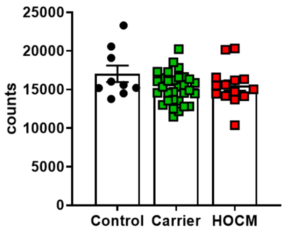

Metabolite 14
sn-glycero-3-Phosphoethanolamine
Glycerylphosphorylethanolamine

Androstane steroids

Metabolite 1

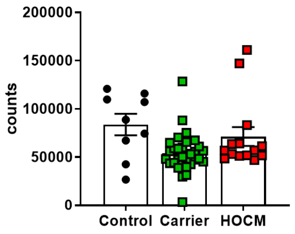

Metabolite 1
19-Hydroxyandrost-4-ene-3,17-dione
19-Oxotestosterone
7a-Hydroxyandrost-4-ene-3,17-dione
11b-Hydroxyandrost-4-ene-3,17-dione
16a-Hydroxyandrost-4-ene-3,17-dione
4-Methoxy-17beta-estradiol
2-Hydroxyestradiol-3-methyl ether

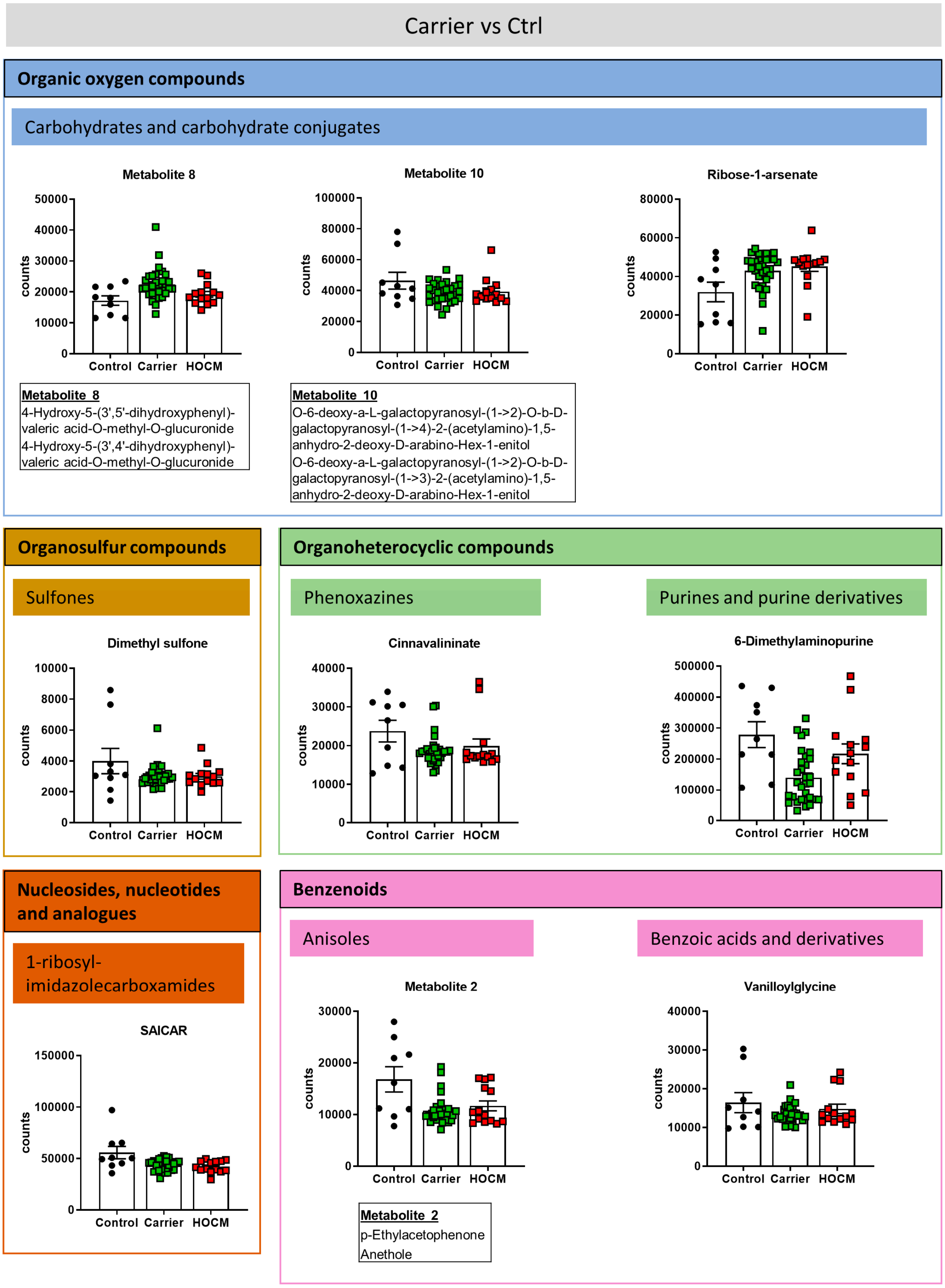

Figure S3: Bar graphs with individual data points of the top 30 most important metabolites in distinguishing the Carrier vs Ctrl group. Data from the third group is included in all graphs.

Carrier vs HOCM

Organic acids and derivatives

Amino acids, peptides and analogues

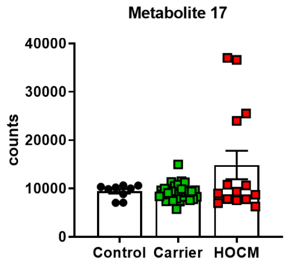

Metabolite 17
Aspartyl-Glutamate
Glutamyl-Aspartate

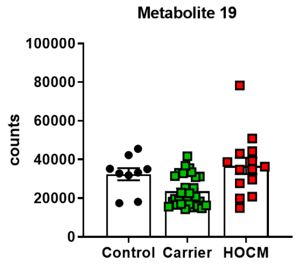

Metabolite 19
Hydroxyprolyl-Valine
Valyl-Hydroxyproline

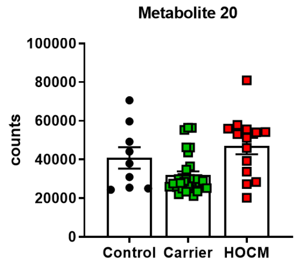

Metabolite 20
Asparaginyl-Tryptophan
Histidinyl-Tyrosine
Tryptophyl-Asparagine
Tyrosyl-Histidine

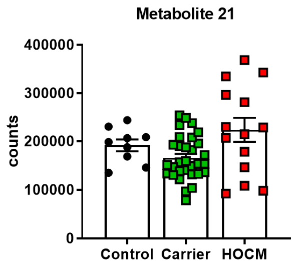

Metabolite 21
Asymmetric dimethylarginine
Symmetric dimethylarginine

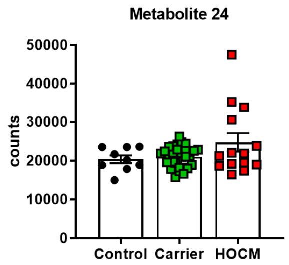

Metabolite 24
Asparaginyl-Lysine
Lysyl-Asparagine

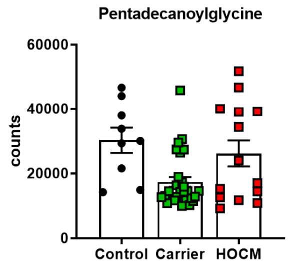

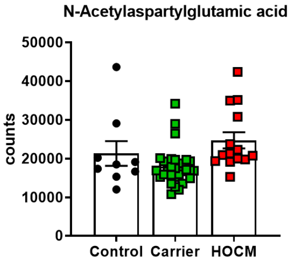

Organic oxygen compounds

Carbonyl compounds

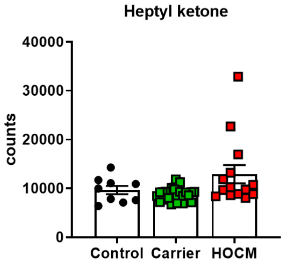

Carbohydrates and carbohydrate conjugates

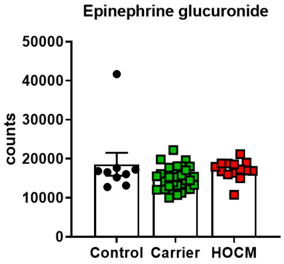

Phenylpropanoids and polyketides

Isoflavans

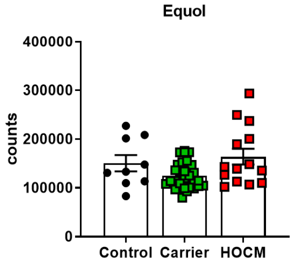

Carrier vs HOCM

Lipids and lipid-like molecules

Fatty acids and conjugates

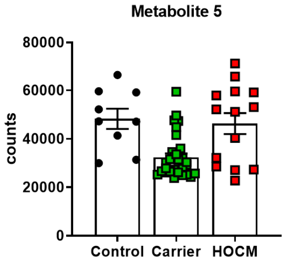

Metabolite 5
11-peroxy-5Z,8Z,12E,14Z-eicosatetraenoate
9-peroxy-5Z,7E,11Z,14Z-eicosatetraenoate

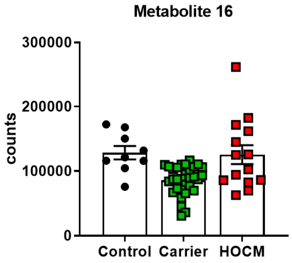

Metabolite 16
Medroxyprogesterone
17-HDoHE
19(20)-EpDPE
16(17)-EpDPE
10-HDoHE
11-HDoHE
16-HDoHE
20-HDoHE
4-HDoHE
7-HDoHE
8-HDoHE
4-Hydroxy-all-trans-retinyl acetate

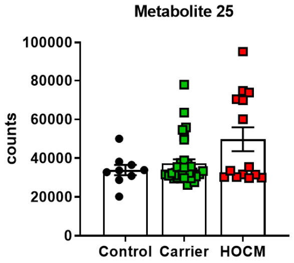

Metabolite 25
Tetracosahexaenoic acid
Tetracosahexaenoic acid, n-3

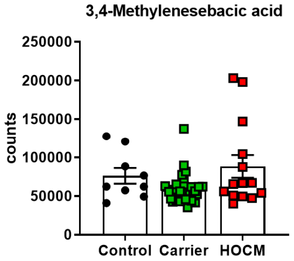

Eicosanoids

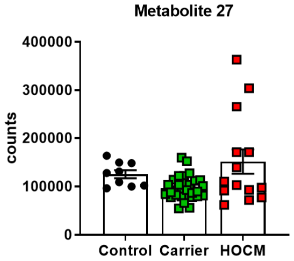

Metabolite 27
15-Keto-13,14-dihydroprostaglandin A2
Prostaglandin J2
Prostaglandin A2
12-Keto-leukotriene B4
Prostaglandin B2
Delta-12-Prostaglandin J2
Leukotriene B5
5-Oxo-6-trans-leukotriene B4
7'-Carboxy-gamma-chromanol
15d PGD2
bicyclo-PGE2
Prostaglandin-c2

Estrane steroids

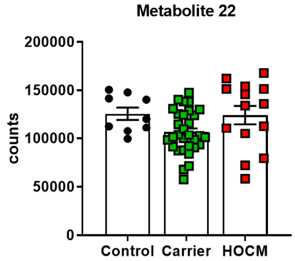

Metabolite 22
Estriol
2-Hydroxyestradiol
16b-Hydroxyestradiol
17-Epiestriol
16,17-Epiestriol
4-Hydroxyestradiol
2-Polyprenyl-3-methyl-6-methoxy-1,4-benzoquinone
4-hydroxystradiol

Lineolic acids and derivatives

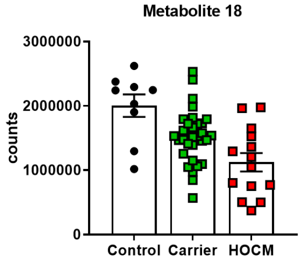

Metabolite 18
MG(0:0/18:3(6Z,9Z,12Z)/0:0)
MG(0:0/18:3(9Z,12Z,15Z)/0:0)
MG(18:3(6Z,9Z,12Z)/0:0/0:0)
MG(18:3(9Z,12Z,15Z)/0:0/0:0)

Tetraterpenoids

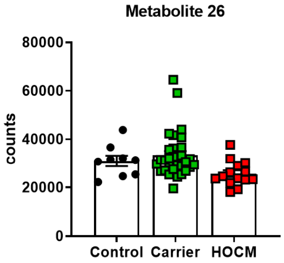

Metabolite 26
3'-Hydroxy-e,e-caroten-3-one
3-Hydroxy-b,e-caroten-3'-one

Cholestane steroids

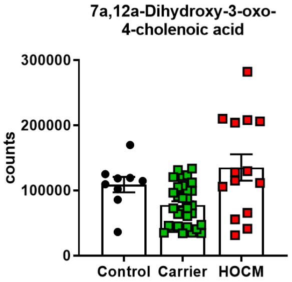

Oxosteroids

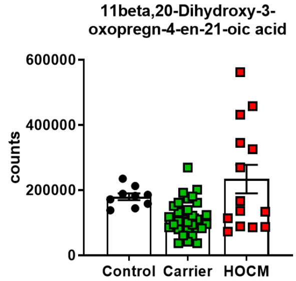

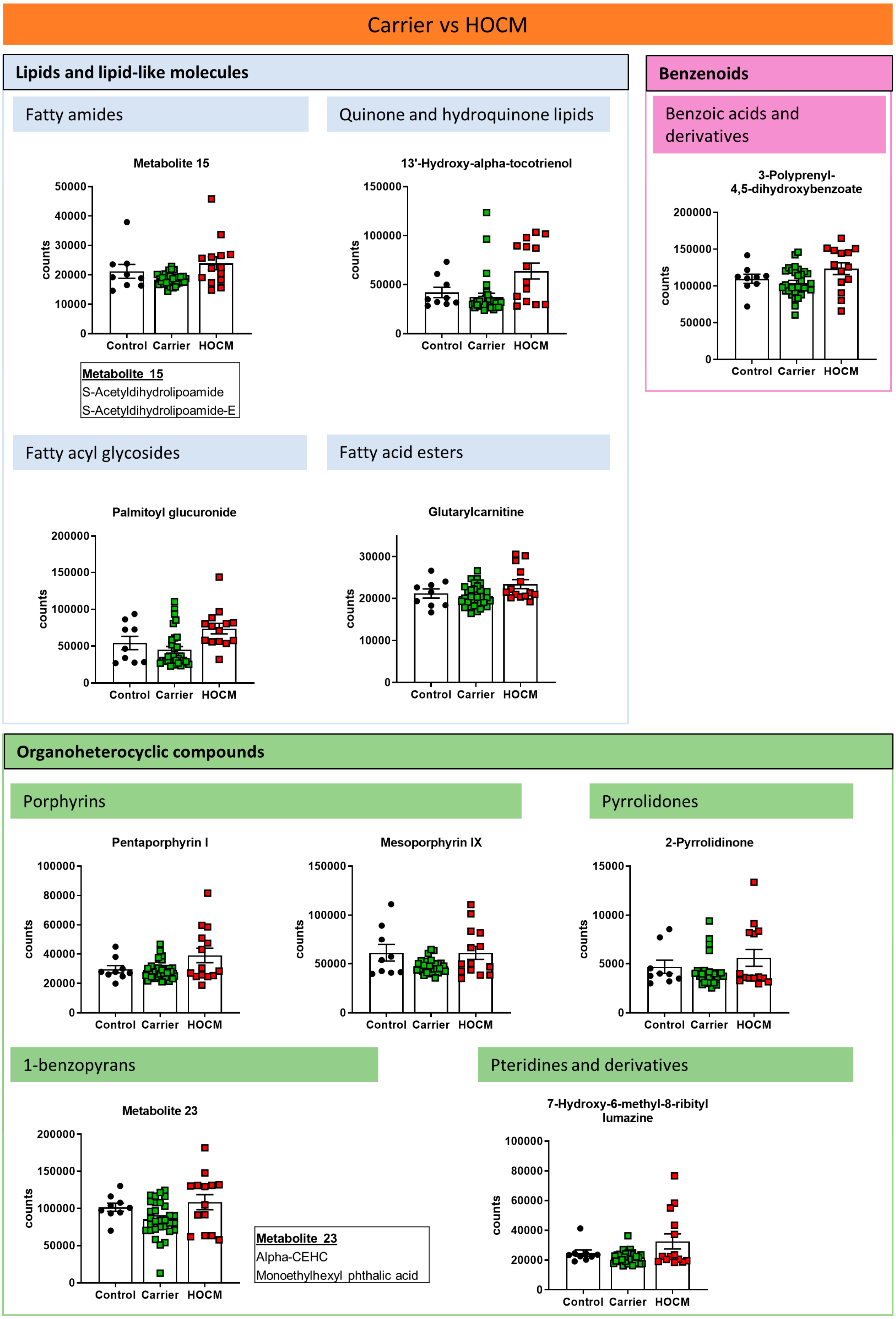

Carrier vs HOCM
Lipids and lipid-like molecules
Fatty amides
Metabolite 15
counts
Control Carrier HOCM
Metabolite 15
S-Acetyldihydrolipoamide
S-Acetyldihydrolipoamide-E
Quinone and hydroquinone lipids
13'-Hydroxy-alpha-tocotrienol
Fatty acyl glycosides
Palmitoyl glucuronide
Fatty acid esters
Glutarylcarnitine
Benzenoids
Benzoic acids and derivatives
3-Polyprenyl-4,5-dihydroxybenzoate
Organoheterocyclic compounds
Porphyrins
Pentaporphyrin I
Mesoporphyrin IX
Pyrrolidones
2-Pyrrolidinone
1-benzopyrans
Metabolite 23
Metabolite 23
Alpha-CEHC
Monoethylhexyl phthalic acid
Pteridines and derivatives
7-Hydroxy-6-methyl-8-ribityl lumazine

Figure S4: Bar graphs with individual data points of the top 30 most important metabolites in distinguishing the Carrier vs HOCM group. Data from the third group is included in all graphs.

HOCM vs Ctrl

Organic acids and derivatives

Amino acids, peptides and analogues

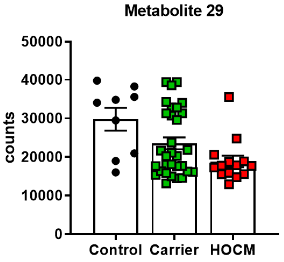

Metabolite 29
Lysyl-Glutamine
Lysyl-Gamma-glutamate
Gamma-glutamyl-Lysine
Glutaminyl-Lysine

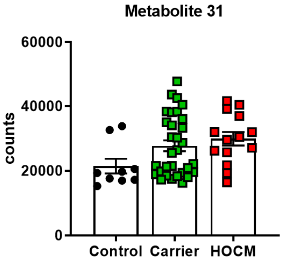

Metabolite 31
Cysteinyl-Leucine
Isoleucyl-Cysteine
Leucyl-Cysteine
Cysteinyl-Isoleucine

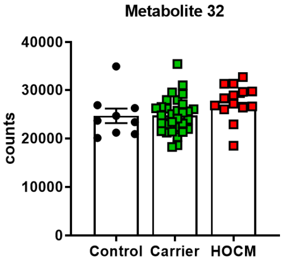

Metabolite 32
Tyrosyl-Glutamine
Tyrosyl-Gamma-glutamate
gln-tyr
Glutaminyl-Tyrosine

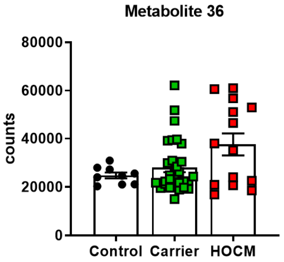

Metabolite 36
Tyrosyl-Cysteine
Cysteinyl-Tyrosine

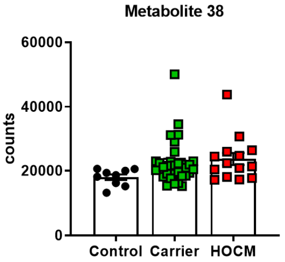

Metabolite 38
Histidinyl-Threonine
Threoninyl-Histidine
2-(3-Carboxy-3-aminopropyl)-L-histidine

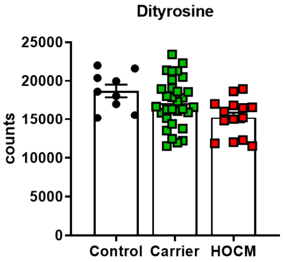

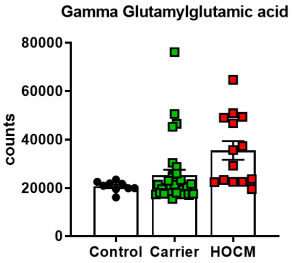

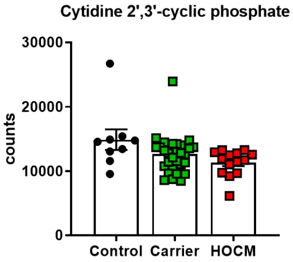

Nucleosides, nucleotides and analogues

Purine deoxyribonucleotides

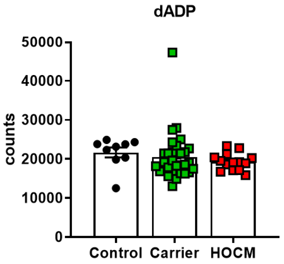

Glycinamide ribonucleotides

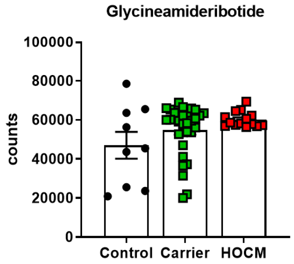

1-ribosyl-imidazolecarboxamides

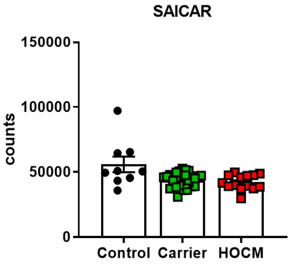

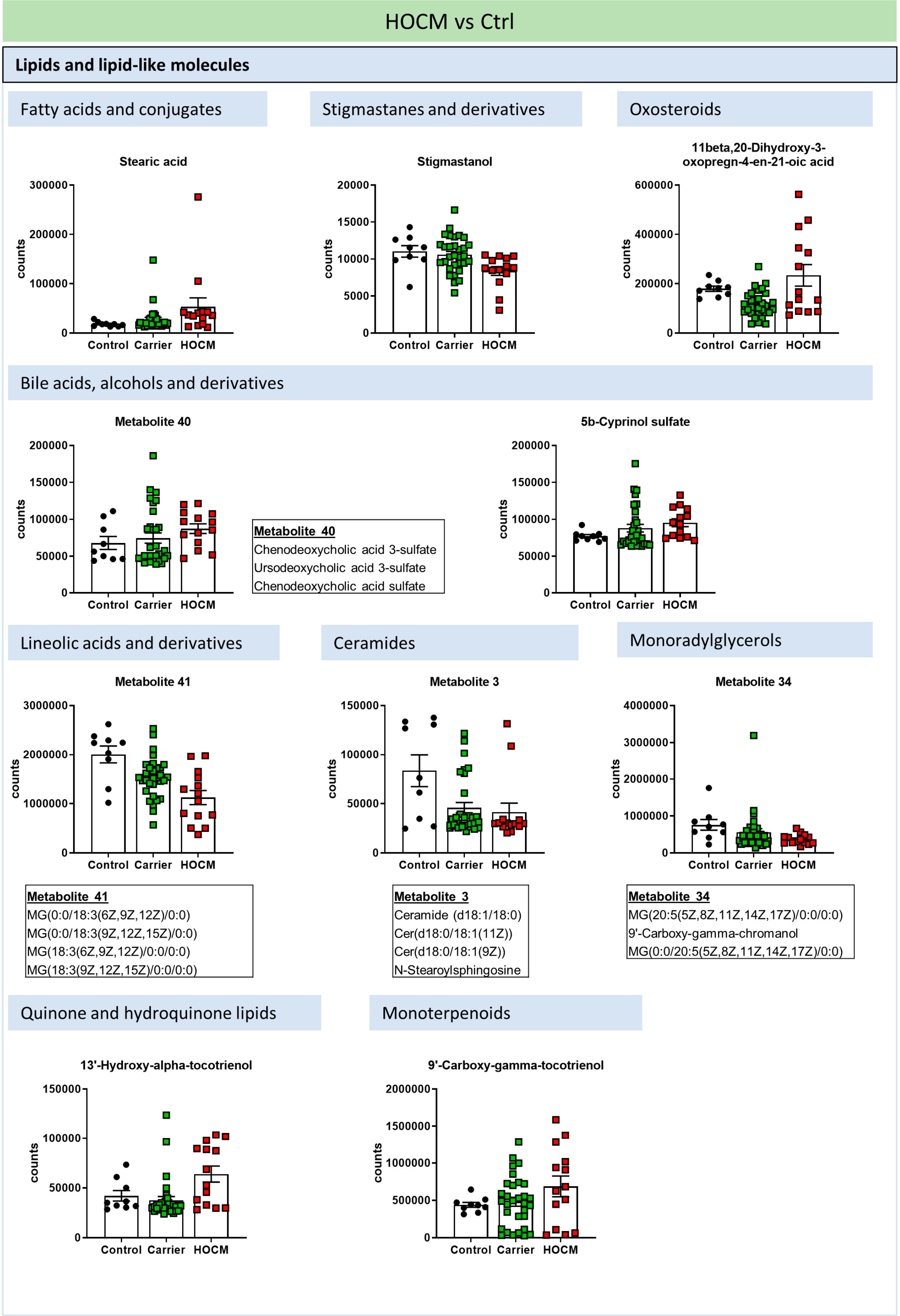
HOCM vs Ctrl
Lipids and lipid-like molecules
Fatty acids and conjugates
Stearic acid
Stigmastanes and derivatives
Stigmastanol
Oxosteroids
11beta,20-Dihydroxy-3-oxopregn-4-en-21-oic acid
Bile acids, alcohols and derivatives
Metabolite 40
Metabolite 40
Chenodeoxycholic acid 3-sulfate
Ursodeoxycholic acid 3-sulfate
Chenodeoxycholic acid sulfate
5b-Cyprinol sulfate
Lineolic acids and derivatives
Metabolite 41
Metabolite 41
MG(0:0/18:3(6Z,9Z,12Z)/0:0)
MG(0:0/18:3(9Z,12Z,15Z)/0:0)
MG(18:3(6Z,9Z,12Z)/0:0/0:0)
MG(18:3(9Z,12Z,15Z)/0:0/0:0)
Ceramides
Metabolite 3
Metabolite 3
Ceramide (d18:1/18:0)
Cer(d18:0/18:1(11Z))
Cer(d18:0/18:1(9Z))
N-Stearoylsphingosine
Monoradylglycerols
Metabolite 34
Metabolite 34
MG(20:5(5Z,8Z,11Z,14Z,17Z)/0:0/0:0)
9'-Carboxy-gamma-chromanol
MG(0:0/20:5(5Z,8Z,11Z,14Z,17Z)/0:0)
Quinone and hydroquinone lipids
13'-Hydroxy-alpha-tocotrienol
Monoterpenoids
9'-Carboxy-gamma-tocotrienol
counts
Control Carrier HOCM

HOCM vs Ctrl

Lipids and lipid-like molecules

Diradylglycerols

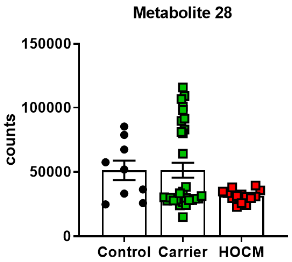

Metabolite 28
DG(16:0/15:0/0:0)
DG(15:0/0:0/16:0)
DG(15:0/16:0/0:0)

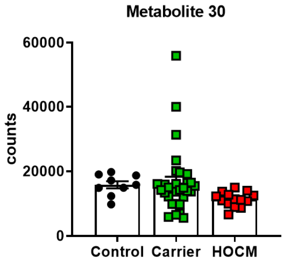

Metabolite 30
DG(15:0/14:0/0:0)
DG(14:0/0:0/15:0)
DG(14:0/15:0/0:0)

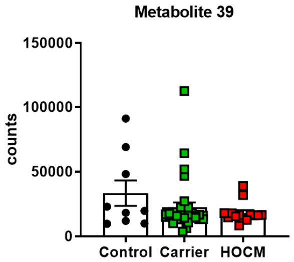

Metabolite 39
DG(14:1(9Z)/20:0/0:0)
DG(16:0/18:1(11Z)/0:0)
DG(16:0/18:1(9Z)/0:0)
DG(16:1(9Z)/18:0/0:0)
DG(18:0/16:1(9Z)/0:0)
DG(18:1(11Z)/16:0/0:0)
DG(18:1(9Z)/16:0/0:0)
DG(20:0/14:1(9Z)/0:0)
DG(20:1(11Z)/14:0/0:0)
DG(14:0/0:0/20:1n9)
DG(16:0/0:0/18:1n7)
DG(16:0/0:0/18:1n9)
DG(18:0/0:0/16:1n7)
DG(20:0/0:0/14:1n5)
DG(14:0/20:1(11Z)/0:0)

Organic oxygen compounds

Carbohydrates and carbohydrate conjugates

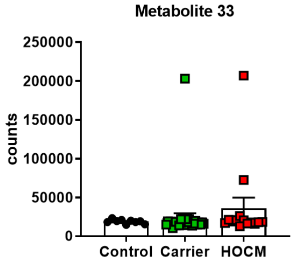

Metabolite 33
N-Acetyl-a-neuraminic acid
N-Acetylneuraminic acid

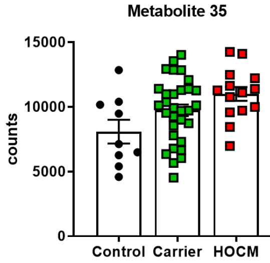

Metabolite 35
5-(3',4',5'-trihydroxyphenyl)-gamma-valerolactone-O-methyl-5'-O-glucuronide
5-(3',4',5'-trihydroxyphenyl)-gamma-valerolactone-O-methyl-4'-O-glucuronide

Benzenoids

Anisoles

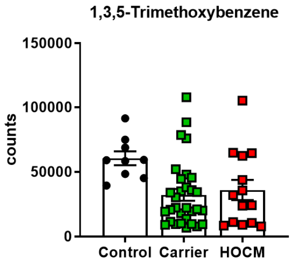

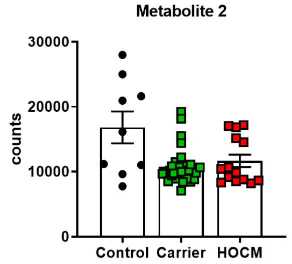

Metabolite 2
p-Ethylacetophenone
Anethole

Organoheterocyclic compounds

Pterins and derivatives

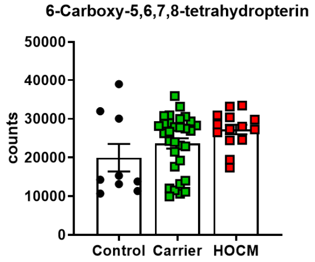

Hydroxyindoles

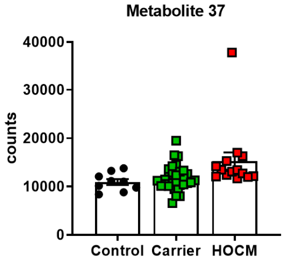

Metabolite 37
5-Hydroxyindoleacetaldehyde
Indoleacetic acid

Figure S5: Bar graphs with individual data points of the top 30 most important metabolites in distinguishing the HOCM vs Ctrl group. Data from the third group is included in all graphs.
